# Mycobacterium susceptibility to ivermectin by inhibition of eccD3, an ESX-3 secretion system component.

**DOI:** 10.1371/journal.pcbi.1012936

**Published:** 2025-04-17

**Authors:** Ana Laura Granados-Tristán, Mauricio Carrillo-Tripp, Carlos Eduardo Hernández-Luna, Aldo Herrera-Rodulfo, Laura Adiene González-Escalante, Ana Leticia Arriaga-Guerrero, Beatriz Silva-Ramírez, Brenda Leticia Escobedo-Guajardo, Roberto Mercado-Hernández, Mario Bermúdez de León, Katia Peñuelas-Urquides

**Affiliations:** 1 Departamento de Biología Molecular, Centro de Investigación Biomédica del Noreste, Instituto Mexicano del Seguro Social, Monterrey, Nuevo León, México; 2 Facultad de Ciencias Biológicas, Universidad Autónoma de Nuevo León (UANL), San Nicolás de los Garza, Nuevo León, México; 3 Laboratorio de la Diversidad Biomolecular, Centro de Investigación y de Estudios Avanzados del Instituto Politécnico Nacional, Unidad Monterrey, Apodaca, Nuevo León, México; 4 Departamento de Inmunogénetica, Centro de Investigación Biomédica del Noreste, Instituto Mexicano del Seguro Social, Monterrey, Nuevo León, México; Shiraz University, IRAN, ISLAMIC REPUBLIC OF

## Abstract

Drug-resistant tuberculosis is a pressing global health issue that requires the development of new drugs or the identification of new therapeutic targets. The ESX-3 secretion system is essential for the *Mycobacterium tuberculosis* growth and plays a role in iron/zinc homeostasis and virulence. The aim of this study was to evaluate the quaternary interface of EccD3, a component of the ESX-3 secretion system, and to evaluate the association of an *eccD3* mutant with drug resistance. The molecular structures of EccD3 protein and other ESX-3 secretion system proteins of the *M. tuberculosis* were predicted based in homology with the *Mycolicibacterium smegmatis* tertiary protein structures. According to the *in silico* results, selamectin, avermectin, ivermectin, and moxidectin were selected as prospective drugs. Selamectin and moxidectin had favorable ΔG values for the EccB3 and EccD3 dimer interfaces, whereas the ESX-3 Protomer 1 interface had the best ΔG + with avermectin, ivermectin, and moxidectin. Furthermore, ivermectin susceptibility increased when the *eccD3* gene was inhibited using CRISPRi in *M. smegmatis*. Blockage of EccD3 increased the ivermectin action, but the modest changes observed may be explained by the compensatory mechanisms or other ivermectin targets in absence of this Esx3 component. Further *in vitro* and preclinical studies are required to validate our findings.

## Author summary

Reducing drug-resistant tuberculosis is a priority within the framework of the health and well-being sustainable development goals, which require the development of new drugs or identification of novel therapeutic targets. Proteins serve diverse functions in cells, with some being crucial for mycobacterial growth, virulence, and pathogenesis. Mycobacteria employ various systems to secrete proteins essential for proper cellular function. The ESX-3 secretion system is vital for *Mycobacterium tuberculosis* survival and plays a role in metal homeostasis and virulence. This study conducted an *in silico* evaluation of EccD3, ESX-3 secretion system component, potential as a drug target and assessed the growth of a mutant lacking of *eccD3* in the presence of drugs. Simulations of interactions between the EccD3 and others ESX-3 secretion system amino acid interfaces and 34 drugs revealed that selamectin, avermectin, ivermectin, and moxidectin exhibited the most favorable binding potential. Furthermore, when evaluating the growth of an *eccD3 M. smegmatis* mutant in the presence of ivermectin, an increase in drug susceptibility was observed. However, this change in susceptibility was modest, potentially due to compensatory mechanisms employed by mycobacteria in the absence of *eccD3.* Further studies are warranted to explore these findings in greater depth.

## Introduction

Tuberculosis (TB), caused by *Mycobacterium tuberculosis*, is one of the leading causes of death worldwide. Over 10.8 million incident cases occurred in 2023 [1]. However, the efficacies of first-line regimens that combine isoniazid, rifampicin, ethambutol, and pyrazinamide have decreased due to the emergence of drug-resistant strains [2]. Patients with drug-resistant TB (DR-TB) require treatment with second-line therapeutics, such as fluoroquinolones, or bacteriostatic drugs. Second-line drugs are more toxic and require longer treatment durations [2]. The identification of biomolecular markers or novel therapeutic targets are crucial in the treatment and control of DR-TB, as is the repurposing of existing drugs or the discovery or design of new compounds. Studies of DR-TB biomolecular markers have been performed showing different potential genes to explore therapeutic targets [3–6] or new compounds [7]. The ESX-3 secretion system is essential for *M. tuberculosis in vitro* growth, however this phenotype can be restored by adding iron in the culture [8]. Furthermore, ESX-3 plays a role in iron/zinc homeostasis [9, 10] and virulence [11, 12]. The ESX-3 locus, which is highly conserved in mycobacterial genomes, consists of several genes, including the *ecc* genes (*eccA3, eccB3, eccC3*, *eccD3*, and *eccE3*), encoding ATP-domains and transmembrane domain proteins, whose are the focus of this study. Other genes in the locus include the *esx* genes (*esxG* and *esxH*) and *pe/ppe* genes (*PE4* and *PPE5*), which encode secreted proteins, the *espG3* gene, which encodes a chaperone protein, and the *mycP3* gene, which encodes a membrane-anchored subtilisin-like serine protease [13]. Two similar protomers of ESX-3 secretion machinery that form a stable dimer have been identified recently in *Mycolicibacterium smegmatis* [12,14,15]. Each protomer is composed of single copies of EccB3, EccC3, and EccE3 proteins, and only EccD3 shows two copies. There is no experimental evidence that MycP3 is associated with the core protomer [14, 15]. The ESX-3 secretion system characteristics described in literature suggest that ESX-3 components are potential drug targets [16–18]. Besides, the *esxG* and *esxH* genes, codifying to secreted protein by ESX3 secretion system were associated with DR-TB [3,4,6]. The EccD3 dimer is positioned in ESX-3 complex in a way that interacts with the membrane and the cytosol, and its disruption may cause a system destabilization [14, 15].

Drug development typically spans about 12 to 15 years and consumes vast economic resources [19]. However, computer-aided drug discovery (CADD) may reduce time and cost requirements [20]. *In silico* molecular docking studies predict the interaction quaternary complex and the change in binding free energy (ΔG) of a given drug-target pair. This method facilitates protein drug target screening [21]. Moreover, drug repurposing is considered a shortcut for drug development because it considers the potential use of approved drugs originally intended for other indications [22].

The targeting of bacterial secretion systems represents a viable therapeutic strategy, supported by multiple mechanistic considerations. Recent studies provide evidence that bacterial secretion systems, e.g. the Type VI secretion system T6SS [23] and others, are inherently dynamic rather than static, undergoing substantial conformational changes during the secretion process. These conformational shifts expose different interface regions, creating opportunities for drug intervention even in fully assembled complexes. Furthermore, bacterial secretion systems require continuous maintenance of their quaternary structure through protein turnover and membrane dynamics, necessitating regular component replacement. The targeting of critical interface regions, which structural studies have identified as essential for complex stability [24], provides strategic intervention points where drug binding could destabilize the entire structure. This approach mirrors successful strategies employed against other biological oligomeric systems [25, 26] and membrane protein complexes, including the bacterial divisome [27], establishing a precedent for the therapeutic targeting of protein-protein interfaces in bacterial membrane complexes.

Therefore, the aim of this study was to evaluate EccD3 dimer interface as target for 23 anti-TB and 11 repurposing drugs (34 ligands in total), based on a protein-protein interaction (PPI) inhibitor approach. The *in silico* results were validated using a *M. smegmatis* mutant strain with the *eccD3* gene inhibited. In addition, other quaternary interfaces and ATPase domain of ESX3 secretion system were evaluated as drug targets. For generating the inhibition system, the CRISPR interference (CRISPRi) system [28] was used.

## Results

To evaluate EccD3 protein and the ESX3 quaternary interfaces as drug targets, an *in silico* molecular docking analysis between the proteins and the 23 anti-TB drugs plus 11 repurposing drugs was performed. The structural docking results are accessible in the MDdb Science Gateway at http://md-db.org with entry ID 690005 [24].

**Fig 1:**
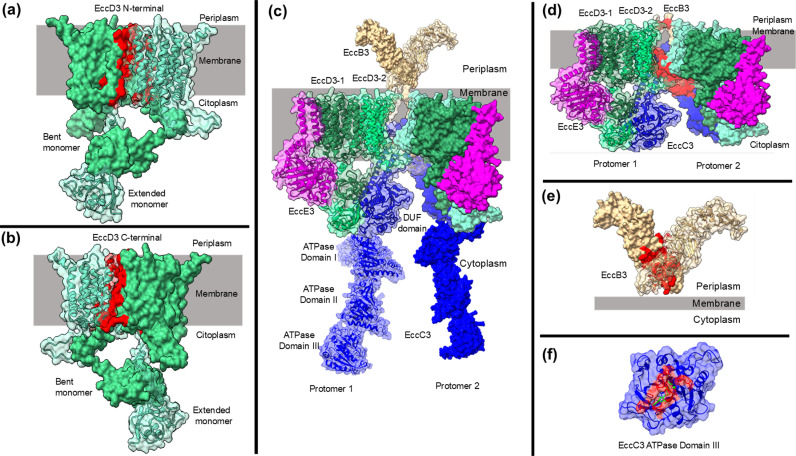
*M. tuberculosis* EccD3 and ESX-3 secretion system models and drug binding site search space. (a) and (b) N- terminal and C-terminal at the quaternary interfaces of EccD3 dimer. (c) Quaternary structure of *M. tuberculosis* of ESX-3 secretion system, using homology models of proteins. EccD3-1 represents bent monomer, EccD3-2 represents extended monomer, DUF represents EccC3 domain of unknown function. (d) Quaternary interface of protomer 1 and protomer 2. (e) Quaternary interface of periplasmic EccB3 dimer. (f) Active drug binding site of ATP in the EccC3 ATPase III domain (1-PDB ID: 6J17) [29]. Quaternary interface amino acids defining the search space are shown in red.

The homology model of the EccD3 dimer composed by the extended and bent monomer was generated (Fig 1a and 1b). Furthermore, the quaternary structure of *M. tuberculosis* ESX-3 secretion system Protomer 1 and Protomer 2, composed of EccB3, EccC3, EccD3-extended monomer, EccD3-bent monomer, and EccE3, is shown in Fig 1c. The stereo-chemical quality of the two protomers was quantified with the MolProbity scoring (S1 Table).

### Identification of drug binding site search space of ESX-3 secretion system proteins

To focus the sampling volume space to those regions that are crucial in quaternary structure formation, we defined our search on the quaternary interfaces between the monomers of EccD3 dimer (Fig 1a and 1b), and two ESX-3 interfaces, the periplasmic monomers of the EccB3 domains (Fig 1e), and between the two protomers (Fig 1d and S2 Table). The ATP-binding site of EccC3 ATPase III domain was also sampled (Fig 1f) as an enzymatic target.

### Identification of potential drug targets for ESX-3 secretion system of *M. tuberculosis*

The EccC3 ATPase domain III has been previously co-crystallized with ATP (1-PDB ID: 6J17) and interacts with magnesium (Mg) [29]. To validate the proposed methodology, a molecular docking control analysis was performed (S1 Fig) using VINA algorithm for all dockings, including evaluations with one ATP molecule with and without a magnesium atom. These results showed ΔG values of -10.4 and -8.8 kcal/mol, respectively, and a structural superposition of the experimental ATP pose with the predicted pose from the molecular docking had a root mean square deviation (RMSD) value of 1.087 Å (S1 Fig). We also found similar interactions between ATP and active site amino acids when comparing experimental data and the two molecular docking evaluations used as controls (S3 Table).

**Fig 2:**
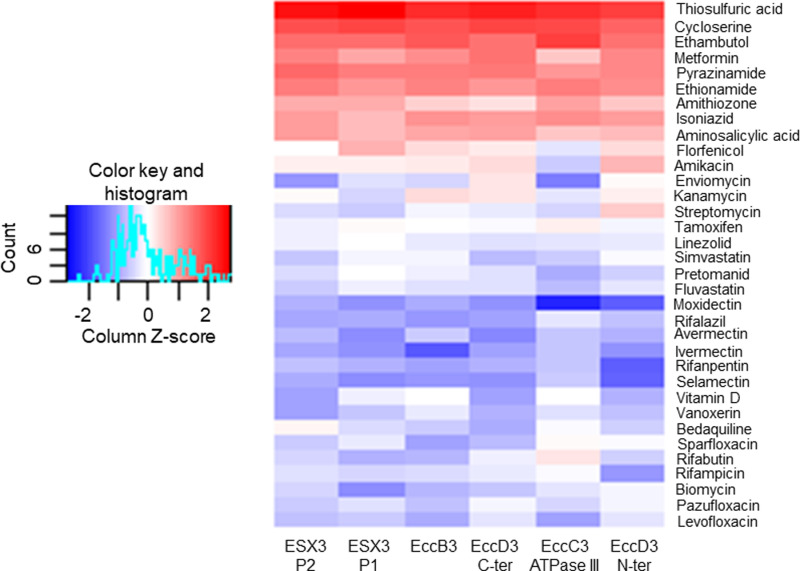
Target-ligand Z-score heat-map based on the lowest ΔG found for each of the six search locations against the 34 drugs. Analysis by columns (independent statistical analysis for each intersection location). Z-score values show in a blue-red gradient, being represented in blue when the Z-score is below the mean and in red when the Z-score is above the mean, if the Z-score approaches to the mean it appears white (S4 Table).

Fig 2 and S4 Table show the results of a column-based Z-score matrix analysis, which compares each target against the entire drug set to identify drugs with the most favorable interactions with a specific target.

**Fig 3:**
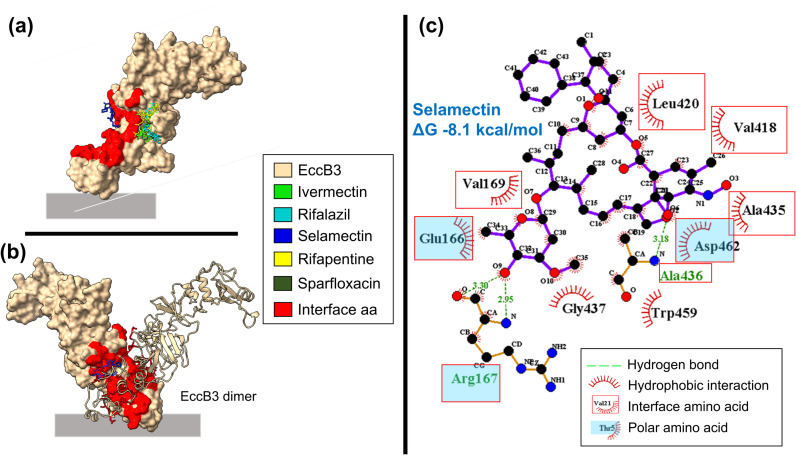
*M. tuberculosis* ESX-3 EccD3 domain. (a) EccD3 N-terminal and EccD3 C-terminal interactions with the nine drugs having Z-score values less than -1. D, Molecular interactions between EccD3 interface residues and (b) selamectin, (c) moxidectin, and (d) rifalazil in 2D diagram. Ligplot was used for 2D map.

**Fig 4:**
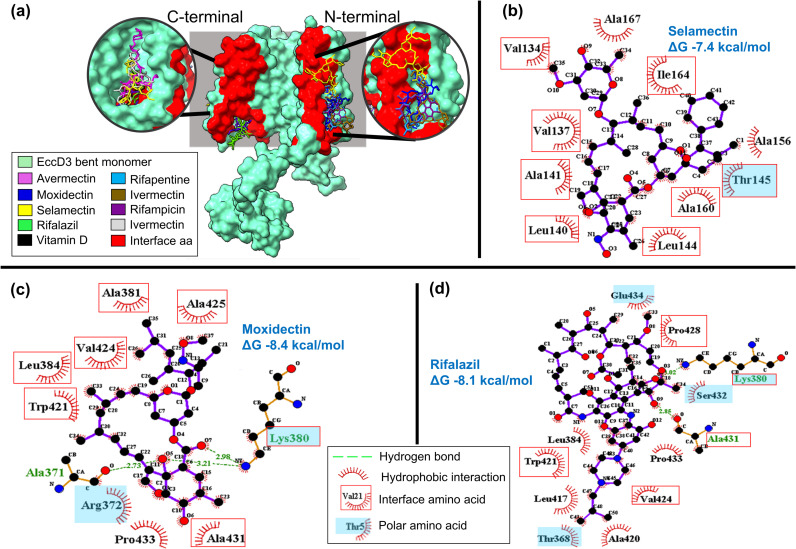
*M*. *tuberculosis* ESX-3 periplasmic domain EccB3. (a) EccB3 interactions with the five drugs having Z-scores values less than -1. (b) EccB3 dimer interface interaction with selamectin. (c) Molecular interactions between EccB3 interface residues and selamectin in 2D diagram. Ligplot was used for 2D map.

**Fig 5:**
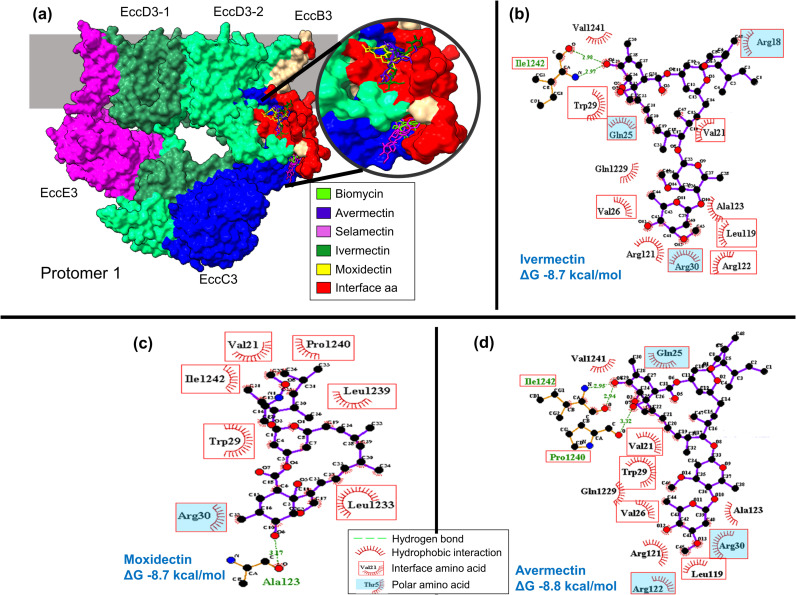
*M. tuberculosis* ESX-3 secretion system protomer 1. (a) Protomer 1 interactions with the five drugs having Z-score values less than -1. Molecular interactions between Protomer 1 interface amino acids with (b) ivermectin, (c) moxidectin, and (d) avermectin in 2D diagram. Ligplot was used for 2D map. EccD3-1, refers to extended monomer and EccD3-2, refers to bent monomer.

The protein-drug pairs with a Z-score value of -1 or less were 1) rifapentine, ivermectin, moxidectin, selamectin, and rifampicin with the EccD3 N-terminal (Fig 3b), 2) avermectin, ivermectin, moxidectin, selamectin, rifalazil, and vitamin D with the EccD3 C-terminal (Fig 3c and 3d), 3) ivermectin, rifalazil, selamectin, rifapentine, and sparfloxacin with EccB3 (Fig 4a), 4) biomycin, avermectin, ivermectin, selamectin, and moxidectin with ESX-3 protomer 1 (Fig 5a), 5) enviomycin, vanoxerine, and vitamin D with protomer 2 (S2 Fig), and 6) enviomycin, moxidectin, and levofloxacin with the EccC3 ATPase III domain (S3 Fig and S4 Table).

Selamectin interacts with EccD3 N-terminal (Fig 3b), showing ΔG value of -7.4 kcal/mol, this complex showed hydrophobic interactions with one polar and seven non-polar interface amino acids (Fig 3b). In addition, selamectin interacts with interface amino acids of EccB3 (Fig 4b and 4c), showing ΔG values of -7.4 kcal/mol. These two drug-protein complexes had similar interactions with non-polar valine, alanine, and leucine interface amino acids (Figs 4c and 3b).

EccD3 C-terminal interface amino acids interacted with moxidectin and rifalazil, showing ΔG values of -8.4 and -8.1 kcal/mol, respectively (Fig 3c and 3d). We observed six hydrophobic interactions with non-polar and one with polar interface amino acids in the EccD3 N-terminal-moxidectin complex (Fig 3c), whereas four hydrophobic interactions with non-polar and one with polar interface amino acids were observed in the EccD3 N-terminal-rifalazil complex (Fig 3d). Alanine 431, tryptophan 421, and valine 424 were interface amino acids that interacted with moxidectin and rifalazil (Fig 3c and 3d).

ESX-3 protomer 1 interface amino acids interacted with avermectin, ivermectin, and selamectin, showing ΔG values of -8.8, -8.7, and -8.7 kcal/mol, respectively (Fig 5a). Interactions with six non-polar and three polar interface amino acids were found in ESX-3-P1-avermectin and -ivermectin complexes (Fig 5d and 5b), whereas interactions with six non-polar and one polar interface amino acids were observed in the ESX-3-P1-moxidectin complex (Fig 5c). Valine 21, tryptophan 29, arginine 30, and isoleucine 1242 were interface amino acids that showed molecular interactions with the three drugs (Fig 5b-d). However, the ESX-3 protomer 2 complexes with enviomycin, vanoxerine and vitamin D, did not show molecular interactions with any interface amino acids (S2 Fig).

Overall, the drugs that interacted with EccD3 interfaces and the others ESX-3 secretion system interfaces having the lowest ΔG values were from the avermectin family (S4 Table); avermectin, ivermectin, moxidectin, and selamectin). Since the complex structure of ivermectin and the glutamate-gated chloride channel of *Caenorhabditis elegans* has been reported (2-PDB ID: 3RIF) [30], we performed a molecular docking analysis between these two as an additional methodological positive control to validate the predicted binding free energy change and structural conformation. The molecular docking pipeline correctly reproduced the glutamate-gated chloride channel and ivermectin complex, showing a ΔG value of -9.4 kcal/mol and an RMSD value of 1.2 Å (S3 Fig). Furthermore, considering that avermetin drugs have a logP>1, i.e. they are somewhat hydrophobic molecules (S5 Table), and that most of the amino acids comprising the sampled protein-protein interface regions are non-polar (Figs 3, 4, 5 and S4). The question arises whether these protein-drug interactions are just driven by nonspecific hydrophobic effect or have some preference for the quaternary interfaces. Hence, we added a molecular docking between all 34 drugs and two physicochemically distinct ESX-3 secretion system solvent-exposed surfaces, the EccD3 mostly-hydrophobic and the EccE3 mostly-hydrophilic surfaces (S5 Fig). On average, avermectin drugs binding energy change to protein-protein interface regions were -8.1, -7.0, -8.4, -8.8, and -8.5 for EccB3, EccC3 N-ter, EccD3 C-ter, ESX-3 P1, and ESX-3 P2, respectively (S4 Table). In contrast, the same avermectin drugs ΔG to solvent-exposed surfaces were, on average, -3.4 and -5.5 for EccD3 and EccE3, respectively (S6 Table). These results indicate a thermodynamic preference of avermectin drugs for protein-protein interfaces over solvent-exposed protein surfaces.

Finally, we checked the catalytic EccC3 ATPase domain III. The most favorable complexes were with moxidectin, envyomicin, and levofloxacin, showing ΔG values of -8.8 kcal/mol, -7.8 kcal/mol, and -7.4 kcal/mol, respectively. We observed interaction between the three drugs and enzyme active site amino acids, however, moxidectin showed the lowest binding free energy change (S6 Fig), comparable to that observed with ATP.

### Effect of eccD3 gene knockdown in *M. smegmatis*

EccD3 is the only protein that forms a homodimer within ESX-3 secretion system structure, therefore inhibition of *eccD3* gene may generate a disruption of the complete structure of the secretion system. The PLJR962-*eccD3*-gRNA plasmid was constructed and characterized by PCR and automatized sequencing; a 186 bp amplified fragment bearing the *eccD3*-gRNA identical sequence was obtained. Subsequently, CRISPRi strain with *eccD3* gene transcription inhibited was generated and characterized by PCR, confirmed by the 186 bp product.

**Fig 6:**
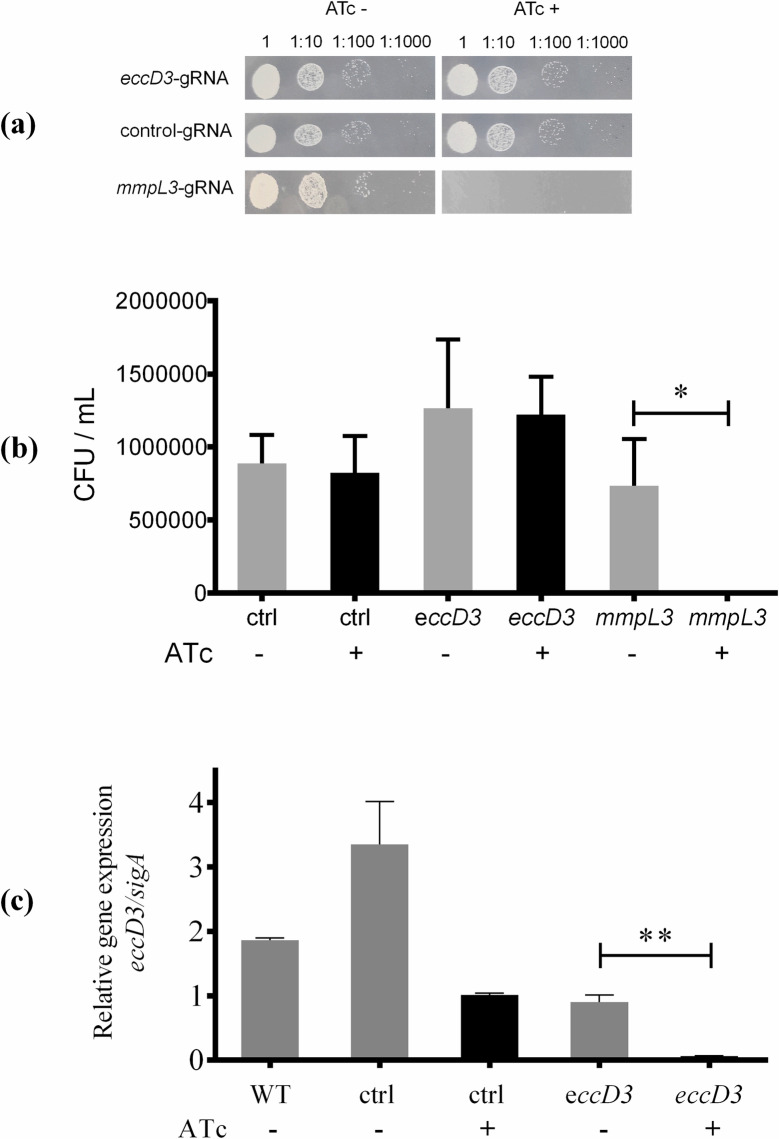
Characterization of CRISPRi *M. smegmatis* strains. (a) Representative image of *M. smegmatis* strains cultures (PLJR962-*eccD3*-gRNA, PLJR962-control-gRNA and PLJR962-*mmpL3*-gRNA) grown in 7H10 medium supplemented with OADC, kanamycin 0.03 µM (20 μg/mL) and without or with ATc. (b) A significant difference in growth was observed in *M. smegmatis* PLJR962-*mmpL3*-gRNA strain without or with ATc, using Student’s t-test (*p= 0.0168*) bars represent the average of the replicates and error bars illustrate standard deviation of the data. (c) Relative expression of *eccD3* gene, average of technical triplicates of each strain without (-) and with (+) ATc (anhydrotetracycline) 0.0002 μM (100 ng/mL). For (b) and (c) *eccD3* corresponds to *M. smegmatis* PLJR962-*eccD3*-gRNA strain, ctrl corresponds to *M. smegmatis* PLJR962-control-gRNA strain, WT corresponds to *M. smegmatis* wild type strain and *mmpL3* corresponds to *M. smegmatis* PLJR962-*mmpL3*-gRNA. The rod with the asterisk shows significant differences between the relative expression of *eccD3* gene in *M. smegmatis* PLJR962-*eccD3*-gRNA strain using Student’s t-test (*p=0.0062*).

Ivermectin was selected to evaluate the association with ESX-3 secretion system owing to its chemical and physic characteristics (S5 Fig). The *M. smegmatis* PLJR962-*eccD3*-gRNA strain minimum inhibitory concentration (MIC) and growth curve were performed. *M. smegmatis* PLJR962-*eccD3*-gRNA and *M. smegmatis* PLJR962-control-gRNA strains showed no differences in their growth using ATc (Fig 6a and 6b), but in *M. smegmatis* PLJR962-*mmpL3*-gRNA strain, significant difference was observed without or with ATc (Fig 6b). The *eccD3* gene inhibition in *M. smegmatis* PLJR962-*eccD3*-gRNA was evaluated by RT-qPCR assays showing a 92.19-fold inhibition with ATc compared with the same strain without ATc (Fig 6c). The scramble sequence selected as control guide in this study did not align anywhere in the *M. smegmatis* genome in *the in silico* analyses, however in the *in vitro* assays an effect was observed in RT-qPCR results (Fig 6c). The Shapiro-Wilk test results indicated that the RT-qPCR data showed a normal distribution with a *p-*value of 0.0904 and a W statistic value of 0.09.

The resazurin microtiter assay plate method (REMA) was used to determine ivermectin susceptibility. The MIC for *M. smegmatis* wild type was 0.25 μM (256 µg/mL) (S7 Fig), and for *M. smegmatis* PLJR962-*eccD3*-gRNA strain the MIC without ATc was 0.125 μM (128 µg/mL) and with ATc was 0.062 μM (64 µg/mL) (S8a, S8b and S9a Figs), showing an increased in ivermectin susceptibility when *eccD3* gene is inhibited. In the case of *M. smegmatis* PLJR962-control-gRNA strain without and with ATc, the MIC was 0.125 μM (128 µg/mL) (S8c, S8d and S9b Figs).

Growth kinetic curves were evaluated to analyze the changes of *M. smegmatis* PLJR962-*eccD3*-gRNA and *M. smegmatis* PLJR962-control-gRNA strains without and with ATc in presence of different ivermectin concentrations (0.5 μM (512 μg/mL), 0.25 μM (256 μg/mL), 0.125 μM (128 μg/mL), 0.062 μM (64 μg/mL), and 0.031 μM (32 μg/mL)). In 0.5 μM (512 μg/mL), 0.25 μM (256 μg/mL), and 0.125 μM (128 μg/mL) ivermectin doses, *M. smegmatis* PLJR962- *eccD3*-gRNA and *M. smegmatis* PLJR962-control-gRNA strains showed no growth (S10 Fig).

**Fig 7:**
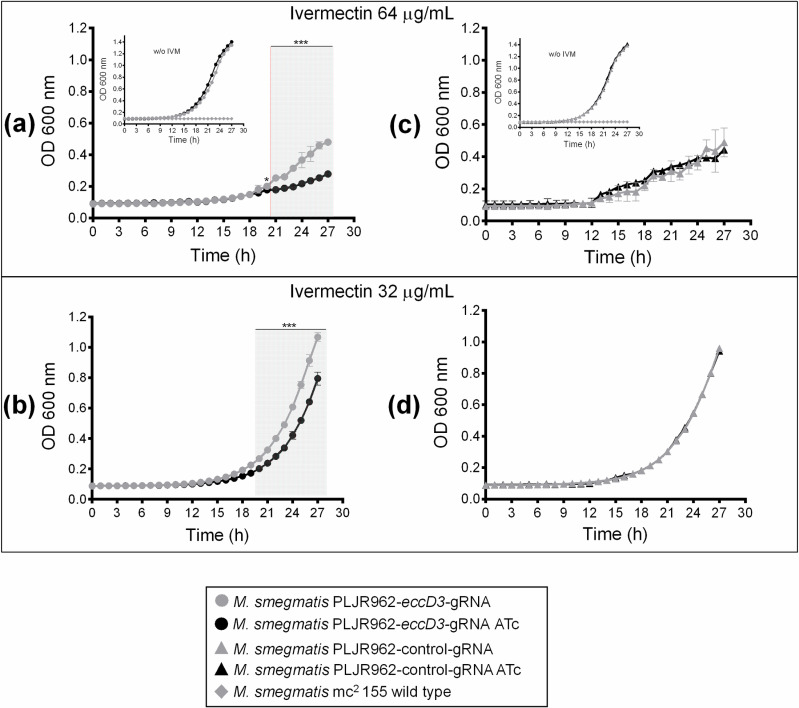
Growth curve of M. smegmatis PLJR962-eccD3-gRNA strain with ivermectin. Growth curves of *M. smegmatis mc*^*2*^ 155 wild type strain (diamonds, inner graph (**a)** and (c), *M. smegmatis* PLJR962-*eccD3*-gRNA strain (circles) (**a)** and (b), and *M. smegmatis* PLJR962-control-gRNA strain (triangles) (c) and (d) without ATc (gray line) and with ATc (black line) 0.0002 μM (100 ng/mL), and with a final concentration of ivermectin of 0.062 μM 64 μg/mL) (a) and (c) and 0.031 μM (32 μg/mL) (b) and (d), and 0.03 μM (20 μg/mL) of kanamycin. In the two ivermectin concentrations tested in *M. smegmatis* PLJR962-*eccD3*-gRNA strain, a significant difference was observed among treatments with and without ATc from 20 to 27 hours. An unpaired two-tailed Student’s *t*-test was used and *p* < 0.001 (***) and *p* <0.05 (*) were obtained (S7 Table and MDdb Science Gateway at http://md-db.org with entry ID 690005 *in-vitro* data). *M. smegmatis* mc^2^ 155 wild type growth curve were included in the inner graph. Each assay was performed with technical triplicates showing the average data with the standard deviation as error bars.

On the other hand, in 64 μg/mL (0.062 µM) and 32 μg/mL (0.031 µM) ivermectin concentrations, a significant difference was found when hourly reads were analyzed with Student *t*-test comparing *M. smegmatis* PLJR962-*eccD3*-gRNA strain when grown without and with ATc (Fig 7a and 7b). Results showed an increase of ivermectin susceptibility when *eccD3* was inhibited (kinetic growth curve with ATc (Fig 7a and 7b). For *M. smegmatis* PLJR962-control-gRNA strain, no differences of bacterial growth were observed (Fig 7c and 7d).

In addition, REMA plated method was used to determine minimum inhibitory concentration for first- and second-line drugs in *M. smegmatis* strains where rifampicin showed a decrease in susceptibility, whereas linezolid showed an increased in susceptibility in *M. smegmatis* PLJR962-*eccD3*-gRNA strain (Table 1).

**Table 1:** Minimum inhibitory concentration of antituberculosis drugs.

Minimum inhibitory concentration µg/mL					
	*M. smegmatis* strains				
Drugs	Wild type	*eccD3*	*eccD3* ATc	Control	Control ATc
Isoniazid	32	16	16	32	32
Rifampicin	6.4	1.6	3.2	1.6	1.6
Levofloxacin	0.125	0.06	0.06	0.06	0.06
Moxifloxacin	0.125	0.125	0.125	0.125	0.125
Linezolid	2	2	1	2	2

Wild type refers to *M. smegmatis* mc^2^ 155 wild type strain, *eccD3* refers to *M. smegmatis* PLJR962-*eccD3*-gRNA strain, and Control refers to *M. smegmatis* PLJR962-control-RNA strain. ATc refers with anhydrotetracycline.

**Fig 8:**
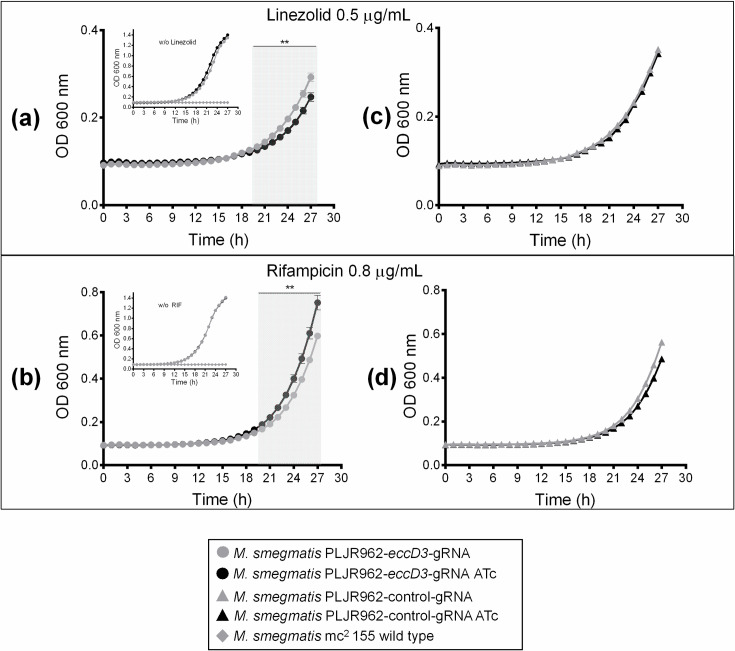
Growth curve of *M. smegmatis* PLJR962-eccD3-gRNA strain with linezolid and rifampicin. Growth curves of *M. smegmatis mc*^*2*^ 155 wild type strain (diamonds, inner graph a and b), *M. smegmatis* PLJR962-*eccD3*-gRNA strain (circles) (a) and (b) without (gray line) and with (black line) ATc 0.0002 μM (100 ng/mL), and *M. smegmatis* PLJR962-control-gRNA strain (triangles) (c and d) without (gray line) and with (black line) ATc 0.0002 μM (100 ng/mL), and with a final concentration of linezolid of 0.001 µM (0.5 μg/mL) (a), and with a final concentration of rifampicin of 0.03 μM (0.8 μg/mL) (b), and (20 μg/mL) of kanamycin. A significant difference was observed in the linezolid and rifampicin concentrations in *M. smegmatis* PLJR962-*eccD3*-gRNA strain without or with ATc from 20 to 27 hours. An unpaired two-tailed Student’s *t*-test was used and *p* < 0.01 (**) were obtained (S8 Table and MDdb Science Gateway at http://md-db.org with entry ID 690005 *in-vitro* data). *M. smegmatis* mc^2^ 155 wild type growth curve were included in the inner graph. Each experiment was performed in technical triplicates showing the average data with the standard deviation.

Growth kinetic curves were evaluated to analyze the changes of *M. smegmatis* PLJR962-*eccD3*-gRNA and *M. smegmatis* PLJR962-control-gRNA strains without and with ATc in presence of different drug concentrations based in the obtained MICs as follows: linezolid (2 µg/mL, 1 µg/mL, and 0.5 µg/mL) and rifampicin (3.2 µg/mL, 1.6 µg/mL, and 0.8 µg/mL) and. When 0.5 μg/mL linezolid concentration was used, an increase of susceptibility when *eccD3* was inhibited (kinetic growth curve with ATc (Fig 8a) was observed. For *M. smegmatis* PLJR962-control-gRNA strain, no differences of bacterial growth were observed (Fig 8b). On the other hand, when 0.8 μg/mL rifampicin concentration was used, a significant difference was observed in *M. smegmatis* PLJR962-*eccD3*-gRNA strain when grown without and with ATc (Fig 8b). Results showed a decrease of rifampicin susceptibility when *eccD3* was inhibited (kinetic growth curve with ATc, Fig 8b). In 0.0038 µM (3.2 µg/mL) and 0.0019 µM (1.6 µg/mL) rifampicin and 0.005 µM (2 µg/mL) and 0.002 µM (1 µg/mL) linezolid doses *M. smegmatis* PLJR962- *eccD3*-gRNA and *M. smegmatis* PLJR962-control-gRNA strains showed no growth (S11 Fig).

## Discussion

The ESX-3 secretion system is required for *in vitro* growth and virulence of *M. tuberculosis*. This system is highly conserved among mycobacteria species [12,18]. EccD3 is the only dimer component of the ESX-3 secretion system. Thus, EccD3 and others ESX-3 components meet the requirements of a potential therapeutic target, particularly through quaternary structure destabilization or ATPase motor inhibition [14, 15]. *In silico* analyses of EccD3 and others ESX-3 proteins quaternary interfaces as drug targets were evaluated and an *M. smegmatis* mutant strain with *eccD3* inhibited gene was generated to evaluate *silico* results. Whereas a quantum mechanics approach may be more suitable for metal-ligand-protein molecular docking analysis [31], some studies have shown that the method employed in this study has an accuracy of more than 73% [32]. To validate our methodology, a docking for ATP and the EccC3 ATPase domain III was performed. The resulting RMSD value between the prediction and the experiment falls within the range of 0-2 Å that is generally considered to indicate molecules with a structure similarity of over 90% [33]. Additionally, an analysis of the docked ATP-protein complex showed interactions with the same amino acids as those reported in the previous experimental study [29]. Based on these results, we can conclude that we successfully validated the molecular docking results presented here.

In this study, we tested five quaternary interface locations within the ESX-3 secretion system as potential targets for the 34 drugs set used, based on the hypothesis that they may disrupt the quaternary structure of ESX-3 and inhibit its function. In recent years, PPI has been a focus in drug design, and PPI inhibitors have shown potential in preventing diseases such as Bcl2/Bax in cancer or Gp120/CCR5 in HIV [34, 35]. Tryptophan, arginine, and tyrosine are present at the five search locations, which aligns with previous research indicating that these amino acids are more likely to be found in PPI hot spots and serve as drug targets [36].

We determined *in silico* that avermectin, ivermectin, moxidectin, and selamectin had the most favorable binding free energy change against the sampled locations in the EccD3 and the ESX3 quaternary interfaces. These drugs are part of the avermectin family and are used as pesticides, anthelmintics, and insecticides. Additionally, avermectin drugs have high molecular weights, around 800 atomic mass units (AMU), and molecules with AMU greater than 500 may be more likely to inhibit protein-protein interactions [37]. The mechanism of action for the avermectin family is thought to involve the drug binding to the glutamate-gated chloride channel in the interface between protein chains, amplifying the effects of glutamate and causing paralysis in invertebrate neuromuscular systems [38]. Our analysis showed that the avermectin drugs interact with the ESX-3 quaternary interfaces in a manner like their interaction with the chloride channel interfaces. Additionally, the ΔG value of ivermectin bound to the glutamate-chloride channel of *C. elegans* had a 1 kcal/mol difference from the binding energy values of avermectin, ivermectin, moxidectin, and selamectin drugs bound to the ESX-3 secretion system, which falls within the range of molecular docking error [39]. Therefore, the binding energy values are similar between the molecular docking control group and the experimental group.

Furthermore, we estimated the biological activities of the avermectin drugs using three database webservers as mentioned in material and methods section (S11, S12 and S13 Tables). Avermectin and ivermectin are more likely to exhibit antibacterial and antibiotic activities than moxidectin and selamectin (S11 Table). In terms of toxicity, moxidectin and selamectin have higher lethal dose 50 (LD50) values than avermectin and ivermectin (S12 Table). Additionally, the four avermectin drugs only demonstrate immunotoxicity but no other types of toxicity (S12 Table). Moreover, avermectin and ivermectin have better water solubility than selamectin and moxidectin. None of the four drugs show blood brain barrier permeability and all drugs have low gastrointestinal absorption (S13 Table).

Adverse effects of avermectin drugs have been reported previously [38]. However, the concentrations of avermectins that have been reported to cause adverse effects are higher than those that have been shown to inhibit the growth of *M. tuberculosis* [40–42]. We used PASSonline, Pro Tox-II, and SwissADME to estimate the biological properties of the avermectin drugs and found that selamectin, moxidectin, avermectin, and ivermectin are considered safe based on these analysis parameters [43–45]. Despite this, further studies and clinical trials are needed to consider the use of avermectins in the treatment of tuberculosis in humans. Studies previously reported *in vitro* inhibition of drug-resistant and drug-sensitive strains of *M. tuberculosis* and other mycobacteria by selamectin, moxidectin, ivermectin, and avermectin, and they suggest that these drugs may be potential candidates for the treatment of DR-TB and other mycobacterial diseases, although further functional analyses are necessary to confirm this hypothesis [40–42,46,47].

Finally, moxidectin had the best ΔG value for the ATP-drug binding site in the ATPase domain III-EccC3. Nonetheless, we suggest analyzing a wider range of drugs to identify a molecule that may serve as a better competitive inhibitor of this ATPase. Other experimental assays are needed to validate these *in silico* results.

Therefore, based on our *in silico* analysis and the previously published research, we propose that avermectin drugs may involve in ESX-3 secretion system *M. tuberculosis* disruption through alteration of its quaternary structure, but the modest changes could suggest compensatory mechanisms or other ivermectin targets by absence of the EccD3 component. Given that the genetic sequence encoding the ESX-3 secretion system is highly conserved among mycobacteria [12,18], we hypothesize that avermectin drugs may have a similar molecular mechanism of action in other mycobacteria.

From the *in silico* results, the effect of inhibition of *eccD3* gene was evaluated experimentally in presence of ivermectin using a strain created *ex profeso:* the *M. smegmatis*-PLJR962-*eccD3*-gRNA strain. CRISPRi knock down system was evaluated using *M. smegmatis*-PLJR962-*eccD3*-gRNA, *M. smegmatis* PLJR962-control-gRNA, and *M. smegmatis*-PLJR962-*mmpL3*-gRNA strains. In *M. smegmatis*-PLJR962-*mmpL3*-gRNA strain used as a control, a significant decrease in bacterial viability was observed in presence of the inductor of CRISPRi system, ATc, because MmpL3 is an essential transporter of the trehalose monomycolic lipid and is essential for *M. smegmatis* viability [48]. In the case of *M. smegmatis*-PLJR962-*eccD3*-gRNA, *M. smegmatis* PLJR962-control-gRNA no differences in bacterial viability were observed when ATc was added. These results show that *eccD3* is not essential for *M. smegmatis* viability, in accordance with literature [49]. However, according to Nath and collaborators, when an *eccD3* knock out *M. smegmatis* strain was generated the growth of the bacteria was decreased [50], whereas in the present study, *eccD3* gene was not completely inhibited, the viability was not noticeably affected. On the other hand, no alteration growth was observed in *M. smegmatis*-PLJR962-control-gRNA strain without or with ATc, as previously reported when similar control strain was used [28,51,52]. The *M. smegmatis* PLJR962-*eccD3*-gRNA strain showed a 92.19-fold of *eccD3* gene inhibition with ATc compared with the same strain without ATc, however an effect was observed in the *M. smegmatis*-PLJR962-control-gRNA strain with ATc. It has been previously described that RNA sequence as control guides can cause off-target interactions that can induce global transcription changes but not interfere with bacterial growth [23].

In this study, the ivermectin MIC of *M. smegmatis* wild type was 256 μg/mL (0.25μM), but some discrepancies were found in the literature, as *M. smegmatis* ivermectin MIC was reported in 8 μg/mL (0.007μM) [40], and in other study the MIC was not found for some clinical isolates of *M. smegmatis* obtained from infected cats with this bacteria were the clinical isolates were grown until 1,024 μg/mL (1μM) [53]. The inhibition of *eccD3* gene caused an increase of ivermectin susceptibility from a MIC of 128 μg/mL (0.125μM) to 64 μg/mL (0.065μM) in *M. smegmatis*-PLJR962-*eccD3*-gRNA strain. Furthermore, the analyses of *M. smegmatis*-PLJR962-*eccD3*-gRNA growth in presence of other drugs showed an increased in rifampicin resistance and an increased in linezolid susceptibility, in accordance, an increase in rifampicin resistance was shown in another study where a knockout of *eccD3* gene in *M. smegmatis* was evaluated [46].

The *in silico* results showed the best interactions of EccD3 and ESX3 secretion system interfaces with avermectin, ivermectin, selamectin and moxidectin. The interface of ESX3 protomer 1 showed interaction with ivermectin and, as contrary expected, *in vitro* results showed an increased sensitivity for ivermectin in the *eccD3* knockdown mutant *M. smegmatis* strain. It was previously reported that the inhibition of *eccD3* gene may cause a cell wall modification [50], therefore, we could suggest that this phenomenon may cause that ivermectin could have major accessibility to the interface of ESX3 secretion system protomer 1 or even to other proteins. In addition, based in the bacteria capacity to module gene expression when associated to antibiotic resistance [54] and its capacity of adverse conditions adaptation [55, 56], we could suggest that inhibition of *eccD3* gene lead the modification in the expression of other genes including related secretion systems as a compensatory mechanism when mycobacteria are exposed to sublethal doses of drugs. In a recent study, a *M. tuberculosis* mutant with *esxG* and *esxH* genes (components of ESX3 gene cluster) deleted, showed growth and virulence inhibition; however, some colonies of *M. tuberculosis* mutants restored virulence and growth *in vivo* and *in vitro* even without iron supplementation. This phenomenon was caused by a mutation in the Rv3058c gene that codify for the transcriptional repressor of *esxR* and *esxS* genes (*esxG-esxH* paralogous) causing the overexpression of these genes showing the *M. tuberculosis* genetic plasticity in overcoming the loss of a key nutrient uptake [57]. Considering the evidence, we observed that it is important to evaluate the global transcriptomic of the *M. smegmatis* PLJR962-*eccD3*-gRNA strain in presence of ivermectin, rifampicin or linezolid to be able to analyze the bacterial response to these drugs as a compensatory mechanism by altering gene expression or their interactions with other genes.

## Conclusion

EccD3 N and C-terminal and ESX3 secretion system domains EccB3 and ESX3-Protomer 1 quaternary interfaces showed favorable molecular interactions with avermectin, ivermectin, moxidectin, and selamectin by *in silico* prediction. Furthermore, *in vitro* assays demonstrated an increase of ivermectin susceptibility when *eccD3* gene was inhibited by 92.19-fold, which means that the blockage of this component, as part of *Mycobacterium* ESX-3 secretion system, could help to increase the ivermectin action on the bacteria. However, the modest susceptibility change to ivermectin may be explained by the compensatory mechanisms governing the bacterial response, that can be observed by further global gene expression analyses combined with mutational experiments against other components of secretion systems.

## Materials and methods

### Protein structure

Three-dimensional (3D) homology models for *M. tuberculosis* EccB3, EccC3, EccD3, and EccE3 proteins from their complete amino acid sequence were built with the SwissModel server (https://swissmodel.expasy.org/) [58]. The sequences of the proteins were obtained by UniProt database with their corresponding entry EccB3 P9WNR3, EccC3 P9WNA9, EccD3 P9WNQ3, and EccE3 P9WJE5. The closest *M. tuberculosis* phylogenetic homolog with available structural data was *Mycolicibacterium smegmatis*, hence, its membrane domain EccB3 (3-PDB ID: 6UMM_3), periplasmic domain EccB3 (4-PDB ID: 6SGY), EccC3 (5-PDB ID: 6UMM_4), EccD3 (6-PDB ID: 6UMM_2), and EccE3 (7-PDB ID: 6UMM_1) proteins as modeling templates were used. Furthermore, the percent identity of ESX3 secretion system and eccD3 nucleotide sequence between *M. smegmatis and M. tuberculosis* were determined being >65% (S14 and S15 Tables). The 3D structure of ATPase III domain of the EccC3 of M. tuberculosis H37Rv with a co-crystallized ATP molecule was obtained from 1-PDB ID: 6J17 [29]. Structural data curation involved deleting solvent and non-complex ions, adding hydrogen atoms, and assigning charges according to the AMBER-ff14SB force field, using UCSF Chimera version 1.14 [59]. Energy minimization was performed in all structures by 10,000 steps of steepest descent (step size of 0.02 Å), followed by 1,000 steps of conjugated gradient (step size of 0.02 Å) with UCSF Chimera [59]. The 3D structure quality was validated using MolProbity server [60] (http://molprobity.biochem.duke.edu/).

### Ligand selection and preparation

Thirty-four drugs as ligands and ATP, as control, were included in this study set, belonging to different chemical classes. Twenty-three were first- and second-line anti-TB drugs [2]. Eleven were selected from drug repurposing studies aimed to identify novel anti-TB treatments, using “*Mycobacterium tuberculosis*, drug repurposing, and tuberculosis” as keywords in PubMed database searching. The selection criteria for drug repurposing studies were drugs which inhibit *M. tuberculosis* growth [40,61,62] (Table 2).

**Table 2:** Ligands includes in this study.

Molecule	CID	Molecule	CID
Isoniazid	3767	Cycloserine	6234
Pyrazinamide	1046	Amithiozone	9568512
Ethambutol	14052	Thiosulfuric acid	24478
Rifampicin	135398735	Biomycin	54677440
Rifapentine	135403821	Enviomycin	135565326
Rifalazil	135431094	Florfenicol	114811
Rifabutin	135398743	Vanoxerine	3455
Amikacin	37768	Metformin	4091
Streptomycin	19649	Vitamin D	5280795
Kanamycin	6032	Simvastatin	54454
Levofloxacin	149096	Tamoxifen	2733526
Sparfloxacin	60464	Fluvastatin	446155
Pazufloxacin	65957	Avermectin	6858006
Ethionamide	2761171	Ivermectin	6321424
Pretomanid	456199	Moxidectin	9832912
Bedaquiline	5388906	Selamectin	9578507
Linezolid	441401	ATP	5957
4-Aminosalicylic acid	4649		

CID, compound identifier of PubChem.

The ATP molecule was used to reproduce the co-crystallized 6J17 quaternary complex as a control of the molecular docking method.

The 3D structure of each ligand was built using UCSF Chimera, based on the PubChem CID and the canonical simplified molecular input line entry system (SMILES) code (https://pubchem.ncbi.nlm.nih.gov) [63]. Missing hydrogen atoms and GASTEIGER partial charges were assigned to each molecule. Energy minimization of the 34 ligands and ATP was performed to all structures through 10,000 steps of steepest descent (step size of 0.02), followed by 1,000 steps of conjugated gradient (step size of 0.02) with UCSF Chimera. The 3D structures were exported in MOL2 format, then imported into AutoDock Tools [64] (https://autodock.scripps.edu/) and transformed into the PDBQT format.

### Molecular docking

Given the focus of this study, ESX-3 secretion system quaternary interfaces were used as the molecular docking sampling space for potential binding sites for the set of ligands. Macromolecular interfaces were identified with the PDBePISA predictor server (http://www.ebi.ac.uk/pdbe/prot_int/pistart.html) [65] and refined based on literature information (S2 Table) [14, 15].

*In silico* molecular docking was performed using the AutoDock Vina software [66]. The quaternary interfaces between EccD3, periplasmic and transmembrane domains EccB3, ESX-3 protomer 1, and ESX-3 protomer 2 proteins, were used as docking targets for the 23 anti-TB and the 11 repurposing drugs. In the case of the EccC3 ATPase III domain, the ATP-binding site was selected as the docking sampling space as a control because of the availability of co-crystallization data (1-PDB ID: 6J17) [29]. Molecular docking simulations were performed with exhaustiveness of search 6, number of binding modes 1, and maximum energy difference of 3 kcal/mol. The sampling box center and dimensions for each protein was generated from PDBePISA results and previous studies [14, 15]. The PDBePISA results highlighted specific interfaces within EccD3 dimer and ESX-3 secretion system, each comprising interface amino acids. These amino acids were visually marked in the protein structures using red coloring to enhance identification. Subsequently, a sampling box was generated, centered to encompass all the interface amino acids within the designated area. In the final step, the identified amino acids were cross-referenced with existing literature on the ESX-3 secretion system to ensure alignment with previous studies [14, 15] (S2 Table).

The docking pipeline was automated using a bash script. To increase the conformational sampling, we performed each docking simulation 10 independent times.

The lowest binding free energy change (ΔG) values for each target-ligand pair were used to create a numerical matrix (S4 Table) to conduct an all-vs-all target-drug analysis. The data was analyzed using the statistical R function heatmap.2; the columns (targets) were scaled so that their average was 0 and standard deviation was 1, resulting in a Z-score heatmap representation of the docking results. Then, two criteria to select target-ligand conformations with therapeutic drug potential were used. First, the complex had to have a Z-score value of -1 or lower. Second, the ligand had to be bound in a quaternary interface region; the conformational sampling box at each search location was built with ample room to completely include all the interface amino acids of interest, leading to the sampling of some neighboring regions during the molecular docking study. Visual inspection was used to discard cases in which the ligand was bound outside the quaternary interface location, even if it had a low ΔG. The molecular interactions of the target-ligand complexes, including hydrogen bonds and hydrophobic interactions, were examined using Ligplot+ version 2.2 [67] and UCSF Chimera X version 1.2.5 [68].

The molecular docking control to support the specificity of the hydrophobic interactions were performed using the same docking pipeline as described above. Drug binding site DogSite predictor (https://proteins.plus) [62] were used to select the bests ESX-3 secretion system drug bindings sites (S5 Fig). Then the best hydrophobic and the best hydrophilic sites were selected. Next steps were performed following the pipeline described above.

### Drug properties.

The SMILES codes were obtained from PubChem. The biological activities were predicted using the PASS platform that is based on a Bayesian algorithm. The output is the probability of activity (Pa) valued between 0 and 1, where 1 indicates that biological activity is certain to occur and 0 is impossible [43] (http://www.way2drug.com/index.php). Furthermore, toxicities were evaluated by the ProTox-II tool, which is based on chemical similarity and the identification of toxic fragments using compounds with known rodent LD50 values. The output is the toxicity probability, which is indicated as active or inactive [45] (https://tox-new.charite.de/protox_II). In addition, the absorption, distribution, metabolism, and excretion (ADME) were predicted by the SwissADME webserver, which is based on different methods such as the BOILED-Egg, iLOPGP, SILICOS-IT, and Bioavailability Radar [44] (http://www.swissadme.ch/index.php).

### Bacterial strains and culture conditions.

*M. smegmatis* mc2 155 (ATCC 700084) strain was cultivated in 7H9 medium (Becton Dickinson and Co, MD, EUA) containing 10% ADC (albumin, dextrose, and catalase) and 0.05% Tween 80, and it was incubated at 37 °C. *Escherichia coli* DH5α strain was cultivated in Luria Bertani broth medium (US Biological Life Sciences) and incubated at 37 °C. For *M. smegmatis* and *E. coli* mutants, kanamycin 0.03 μM (20 µg/mL) (Sigma-Aldrich, MO, USA) was added to the medium.

### CRISPRi knockdown strains.

CRISPRi system was used for transcriptional knockdown of *eccD3* gene in *M. smegmatis* following Rock protocol [28]. The *eccD3*-gRNA was designed and cloned into the PLJR962 plasmid following the protocol described [28]. The *eccD3*-gRNA consists of 23 nucleotides that target the *eccD3* gene sequence, in the *M. smegmatis* genome followed by strong (CC AGAAG) PAM sequence [28]. A 471 bp gBlocks was designed that contains a 247 bp sequence of the PLJR962 plasmid with the *eccD3*-sgRNA sequence selected inside, and surrounding this plasmid sequence, a restriction enzymes sequences were added in the 3’ and 5’ regions (S12 Fig). Then a 183 bp gBlocks sequence with the *eccD3*-gRNA was cloned in PLJR962 using restriction enzymes *EcoRI* and *SalI* (New England Biolabs, MA, USA). The digested-gBlocks was ligated into the digested-PLJR962 using T4 DNA ligase (New England Biolabs, MA, USA) and ATP (1mM) (Invitrogen, MA, USA) at 16°C overnight to generate PLJR962-*eccD3*-gRNA construction. *E. coli* DH5α was transformed with the constructions and grown in solid media containing kanamycin 0.03 μM (20 µg/mL). The PLJR962-*eccD3*-gRNA construction was confirmed using a PCR-based strategy with the *eccD3*-gRNA forward primer and the reverse primer sec 1834 to amplify a 182 bp sequence, furthermore reverse primer sec 1834 was used for sequencing (S13 Table).

Transformed *M. smegmatis* strain with CRISPRi construction was generated using electroporation protocol as indicated in the literature [6] and confirmed *M. smegmatis* PLJR962-*eccD3*-gRNA strain by the PCR-based strategy mentioned above. *M. smegmatis* PLJR962-*mmpL3*-gRNA strain was generated using gRNA proposed in Rock protocol [28], this construction inhibited essential *mmpL3 M. smegmatis* gene (named as CRISPRi positive control). Furthermore, the *M. smegmatis* PLJR962-control-gRNA strain was generated using Genescript (https://www.genscript.com/tools/create-scrambled-sequence) tool to create a scramble sequence for the gRNA design and it is a non-target sequence (named as CRISPRi negative control). The two control strains were generated and characterized with the same protocol used for *M. smegmatis* PLJR962-*eccD3*-gRNA strain.

### CRISPRi evaluation

CRISPRi strains were grown until they reached the log phase and spotted onto 7H10 solid medium containing 10% OADC (oleic acid, albumin, dextrose, and catalase) and kanamycin 0.03 μM (20 µg/mL) with or without the inducer anhydrotetracycline (ATc) 0.0002 μM (100 ng/mL) (Sigma, Aldrich, MO, USA). A volume of 10 µL with approximately 7,000 cells was drop inoculated in the first spot, and subsequent spots were a ten-fold serial dilution (1:10 to 1:1,000). The plates were incubated at 37 °C for 3 days and CFU (colony forming unit) were counted to calculated CFU/mL.

For genotype CRISPRi transcript evaluation, RNA extraction was performed using TRIzol reagent (Invitrogen, CA, USA) and the Fast Prep (MP Biomedicals, CA, USA) instrument following the modifications previously reported [6]. The RNA was treated with DNAse I (New England Biolabs, MA, USA). The RNA concentration and purity were estimated using a NanoDrop 2000 spectrophotometer at a wavelength of 260/280 nm and the RNA integrity was evaluated by electrophoresis in 1% agarose gel. Two µg of RNA were used for cDNA synthesis, using M-MLV reverse transcriptase (Invitrogen, CA, USA) following the manufacturer’s instructions. The functionality of cDNA was evaluated by constitutive sigA gene amplification and was visualized on 1% agarose gel. Quantitative PCR was performed with primers and TaqMan probes (S4 Table) using 7500 Fast Real Time PCR system (Applied Biosystems, CA, USA). The dynamic range curve was established for each probe, testing cDNA dilutions from 1:8 to 1:512. The 1:32 sample dilution was selected to perform the assay. A technical triplicate for each sample was performed and the data were analyzed using the method proposed by Schmittgen and Livak [69].

### CRISPRi strains susceptibility to ivermectin and other drugs

Minimum inhibitory concentration (MIC) for ivermectin of CRISPRi *M. smegmatis* strains was evaluated by the resazurin microtiter assay plate method (REMA.) previously described [6]. The stock ivermectin (Sigma-Aldrich, MO, EUA) solution was diluted in DMSO (Sigma-Aldrich, MO, EUA) from which a working solution was prepared using 7H9 with ADC incubated at 37 °C and thoroughly mixed at 300 rpm at 37 °C. Rifampicin (Sigma-Aldrich, MO, EUA) stock was diluted in DMSO, linezolid (Sigma-Aldrich, MO, EUA) and isoniazid (Sigma-Aldrich, MO, EUA) stocks were diluted in water, levofloxacin (Sigma-Aldrich, MO, EUA) and moxifloxacin (ChenCruz, SantaCruz Bitech, CA, EUA) stocks solutions were diluted in Sodium hydroxide(1N): water (1:10) and from stocks solutions, the working solutions were prepared using 7H9 supplemented with 10% ADC. CRISPRi strains were grown to log phase in 7H9 culture medium containing 10% ADC, kanamycin 0.03 μM (20 µg/mL) and with or without the inducer anhydrotetracycline (ATc) 0.0002 μM (100 ng/mL). *M. smegmatis* wild type was grown using the same conditions as CRISPRi strain but without kan and ATc. Then all cultures were adjusted to 2.7 McFarland turbidity units, then the inoculum was prepared using a 1:1000 culture dilution. To prepare 96-well microtiter plate ivermectin was two-fold serial diluted in 100 μL of 7H9 medium containing ADC for ivermectin concentration range from 4μM (4096 µg/mL) to 0.00097μM (1 µg/mL), rifampicin concentration range was from 51.6 µg/mL to 0.1 µg/mL, isoniazid concentration range from 256 µg/mL to 0.25 µg/mL, linezolid, levofloxacin and moxifloxacin concentrations range from 8 µg/mL to 0.007 µg/mL. The first column was filled with 100 μL corresponding to the double of drug required concentration of ivermectin or the others control drugs, whereas the remaining wells were filled with 50 μL 7H9 medium supplemented with 10% ADC, kanamycin 0.03 μM (20 μg/mL) as selection drug, and ATc 0.0002 μM (100 ng/mL, if required) as the inductor drug. Each CRISPRi strain was evaluated with and without ATc. Serial dilutions were carried out from the first to penultimate column, and with the last column as a control without ivermectin or the other control drugs. Each well was inoculated with 50 μL of *M. smegmatis* strain inoculum as previously mentioned. Control wells with medium with and without kanamycin were included. Technical triplicates were performed from each experiment. The plates were incubated at 37 °C for 40 h with constant agitation, after which, 30 μL of resazurin dye 0.7 µM (0.2 mg/mL) was added to each well. The plates were incubated for 6 additional hours, and MICs were determined as the lowest concentration of antibiotic that showed no visible growth of the bacteria, as indicated by the conversion of resazurin dye color.

To evaluate the bacterial growth in presence of different ivermectin, rifampicin, and linezolid concentrations, growth kinetic curves of *M. smegmatis* strains were carried out. Both *M. smegmatis* wild type and CRISPRi strains were grown in 7H9 medium supplemented with 10% ADC to log phase until reaching 1 McFarland turbidity units. From the 1 McFarland culture, a 100 times dilution was prepared in 7H9 medium suplemented with 10% ADC, kanamycin 0.03 μM (20 μg/mL) as the selection drug, ATc 0.0002 μM (100 ng/mL, if required) as the inductor, and different ivermectin concentrations 0.5 μM (512 μg/mL), 0.25 μM (256 μg/mL), 0.125 μM (128 μg/mL), 0.062 μM (64 μg/mL), and 0.031 μM (32 μg/mL). For rifampicin 3.2 µg/mL, 1.6 µg/mL, and 0.8 µg/mL concentrations were analyzed, whereas linezolid 2 µg/mL, 1 µg/mL, and 0.5 µg/mL concentrations were analyzed. Each CRISPRi strain was evaluated with and without ATc inductor. Each well of microplate was filled with 200 μL of the inoculum, then incubated at 37 °C with constant agitation for 27 h, using the Synergy HT equipment (Biotek, Agilent, CA, USA) to evaluate the absorbance at 600 nm each hour. Technical triplicates of each experiment were performed.

### Statistical analyses

Data obtained by phenotypic effect of CRISPRi evaluation was analyzed using Student’s t-test to compare differences in growth among CRISPRi strains. To analyze genetic expression of *eccD3* gene, the genetic data expression was evaluated with Student’s t-test and the normality distribution of the data was evaluated using Shapiro-Wilk test [70]. The growth kinetic results of *M. smegmatis* strains in the presence of ivermectin, rifampicin and linezolid were evaluated using an unpaired Student’s *t*-test to compare mean curves scores [71, 72]. Results were significant when *p* ≤ 0.05. All statistical analyses were performed using the IBM SPSS Statistics 23 software, Microsoft-Excel, and GraphPad Prism 6.

## Supporting information

S1 Fig EccC3 ATPase domain III structural superposition of the ATP. Experimental pose (red) and the predicted molecular docking pose (green). EccC3 ATPase domain III experimental data 1-PDB ID 6J17 [29].
(DOCX)

S2 Fig *M. tuberculosis* ESX-3 secretion system protomer 2. Protomer 2 interactions with the three drugs having Z-score values less than -1. EccD3-1, refers to extended monomer and EccD3-2, refers to bent monomer.
(DOCX)

S3 Fig Glutamate-gated chloride-channel of *C. elegans* and ivermectin molecular docking. (a) Docking between C chain and D chain glutamate-gated chloride channel of *C. elegans* with ivermectin. Structural superposition of ivermectin-experimental pose (red, 2-PDB ID 3RIF) and ivermectin-docking pose [30]. Molecular interactions in 2D diagram of glutamate-gated chloride channel and ivermectin (b) experimental data, and (c) molecular docking data. The 2D diagrams (c) and (d) represent amino acids of the corresponding glutamate-gated chloride channel C and D chains, respectively. Ligplot was used for 2D map.
(DOCX)

S4 Fig Interface amino acids of the interaction protein-drug. Bars represent the frequency of each type of amino acids of each drug-target. Interface amino acids interactions with selamectin, moxidectin and rifalazil are illustrated.
(DOCX)

S5 Fig EccD3 and EccE3 3D structures. The hydrophobicity of EccD3 (a) and EccE3 (b) structures hydrophilicity is represented in blue while hydrophobicity in red. Drug binding site of EccD3 (c) and EccE3 (d) is shown in red color.
(DOCX)

S6 Fig *M. tuberculosis* ESX-3 EccC3 ATPase domain III. (a) EccC3 ATPase domain III interactions with the three drugs having Z-scores values less than -1 and the ATP molecule as control. Molecular interactions between the EccC3 ATPase domain III interface amino acids with (b) moxidectin, (c) enviomycin, and (d) levofloxacin in 2D diagram. Ligplot was used for 2D map.
(DOCX)

S7 Fig Ivermectin minimum inhibitory concentration (MIC) determination of *M. smegmatis* wild type strain. Resazurin Microtiter Assay plate method with serial dilutions of ivermectin 4μM to 0.03 μM (4096 μg/mL to 8 μg/mL), MIC was determined at 128 μg/mL. Each experiment was performed in technical triplicates shown in rows.
(DOCX)

S8 Fig Ivermectin minimum inhibitory concentration (MIC) determination of M. smegmatis PLJR962-eccD3-gRNA and *M. smegmatis* PLJR962-control-gRNA strains. Resazurin Microtiter Assay plate method with serial dilutions of ivermectin from 0.5 μM to 0.0009 μM (512 μg/mL to 1 μg/mL). Ivermectin MIC of *M. smegmatis* PLJR962-*eccD3*-gRNA strain without (a) or with ATc (anhydrotetracycline 0.0002 μM (100 ng/mL) (b) was determined at 128 μg/mL and 64 μg/mL respectively, and MIC of *M. smegmatis* PLJR962-control*-*gRNA strain without (c) or with ATc 0.0002 μM (100 ng/mL) (d) were observed at 128 μg/mL. Each experiment was performed in technical triplicates shown in rows.
(DOCX)

S9 Fig Ivermectin MIC of M. smegmatis PLJR962-eccD3-gRNA and *M. smegmatis* PLJR962-control-gRNA strains. Graph of relative values of absorbance measured at 605 nm for (a) *M. smegmatis* PLJR962-*eccD3*-gRNA strain and (b) *M. smegmatis* PLJR962-control*-*gRNA strain without or with ATc 0.0002 μM (100 ng/mL). A low level of absorbance corresponds to pink well, whereas high absorbance is for blue wells. IVM (ivermectin) 0.0009 μM to 0.5 μM. Using Synergy HT (BioTek) microplate reader. Each experiment was performed in technical triplicates showing the average and error bars for standard deviation.
(DOCX)

S10 Fig Growth curve of *M. smegmatis* PLJR962-eccD3-gRNA strain with ivermectin. Growth curve of *M. smegmatis* PLJR962-*eccD3*-gRNA strain (circles) and *M. smegmatis* PLJR962-control*-*gRNA strain (triangles) without (gray line) and with (black line) ATc 0.0002 μM (100 ng/mL), with a final concentration of (A) 0.5 μM (512 μg/mL), (B) 0.25 μM (256 μg/mL), and (C) 0.125 μM (128 μg/mL) of ivermectin, and 0.03 μM (20 μg/mL) of kanamycin. Each experiment was performed in technical triplicates showing the average and respective error bars for standard deviation.
(DOCX)

S11 Fig Growth curve of *M. smegmatis* PLJR962-eccD3-gRNA strain with rifampicin and linezolid. Growth curve of *M. smegmatis* PLJR962-*eccD3*-gRNA strain (circles) and *M. smegmatis* PLJR962-control*-*gRNA strain (triangles) without (gray line) and with (black line) ATc 0.0002 μM (100 ng/mL), with a final concentration of (a) 0.0038 μM (3.2 μg/mL) and (b) 0.0019 μM (1.6 μg/mL) of rifampicin, and (c) 0.005 μM (2 μg/mL) and 0.002 μM (1 μg/mL) of linezolid, and 0.03 μM (20 μg/mL) of kanamycin. Each experiment was performed in technical triplicates showing the average and respective error bars for standard deviation.
(DOCX)

S12 Fig Features of the gblock sequence to eccD3-gRNA cloning. Each gblock sequence has a marker above. Random sequences of 5’ and 3’ regions are underlined. Primer sequences and enzyme restrciction sequences are indicated with the corresponce name. The start and the end of the 247 bp PLJR962 plasmid sequence (*eccD3*-gRNA included) are indicated with two slash symbols (//). *eccD3*-gRNA sequence is indicated with bold letters and gray color. Asterisk symbols indicate the restriction enzymes (*EcoRI* and *SalI*) using to clone *eccD3*-gRNA fragment in PLJR962 plasmid.
(DOCX)

S1 Table Structure models of *M. tuberculosis* quality. (DOCX)

S2 Table Binding sites of possible drug interaction in ESX-3 secretion system components. (DOCX)

S3 Table EccC3 ATPase domain III amino acids of the active site bound to ATP. (DOCX)

S4 Table Molecular docking lowest binding energy and Z-scores for antituberculosis drugs on ESX-3 secretion system components. (DOCX)

S5 Table Avermectin drugs properties. (DOCX)

S6 Table Molecular docking lowest binding energy for the 34 drugs and EccD3-hydrophobix and EccE3-hydrophilic non interface regions (*M. tuberculosis*). (DOCX)

S7 Table Student´s t-test results between M. smegmatis PLJR962-eccD3-gRNA and *M. smegmatis* PLJR962-eccD3-gRNA ATc in presence of 64 µg/mL and 32 µg/mL ivermectin. (DOCX)

S8 Table Student´s t-test results between *M. smegmati*s PLJR962-eccD3-gRNA and *M. smegmatis* PLJR962-eccD3-gRNA ATc in presence of 0.5 µg/mL linezolid and 0.8 µg/mL rifampicin. (DOCX)

S9 Table Avermectin drugs biological activities. (DOCX)

S10 Table Avermectin drugs toxicity. (DOCX)

S11 Table Avermectin drugs ADME properties. (DOCX)

S12 Table Identity percent of the alignment between ESX3 secretion system nucleotide sequence *M. smegmatis* and *M. tuberculosis.* (DOCX)

S13 Table Identity percent of the alignment between eccD3 gene nucleotide sequence *M. smegmatis* and *M. tuberculosis.* (DOCX)

S14 Table Sampling box center and dimensions for each protein used for the molecular docking. (DOCX)

S15 Table Primers and probes used in this study. (DOCX)

## References

[1] WHO. Global tuberculosis report. 2023.

[2] Parums DV. Editorial: updates from the World Health Organization (WHO) on global treatment recommendations for drug-susceptible and multidrug-resistant tuberculosis. Med Sci Monit. 2021;27:e934292. doi: 10.12659/MSM.934292 34366429 PMC8362336

[3] Peñuelas-Urquides K, González-Escalante L, Villarreal-Treviño L, Silva-Ramírez B, Gutiérrez-Fuentes DJ, Mojica-Espinosa R, et al. Comparison of gene expression profiles between pansensitive and multidrug-resistant strains of Mycobacterium tuberculosis. Curr Microbiol. 2013;67(3):362–71. doi: 10.1007/s00284-013-0376-8 23649743

[4] González-Escalante L, Peñuelas-Urquides K, Said-Fernández S, Silva-Ramírez B, Bermúdez de León M. Differential expression of putative drug resistance genes in Mycobacterium tuberculosis clinical isolates. FEMS Microbiol Lett. 2015;362(23):fnv194. doi: 10.1093/femsle/fnv194 26454220

[5] Arriaga-Guerrero AL, Hernández-Luna CE, Rigal-Leal J, Robles-González RJ, González-Escalante LA, Silva-Ramírez B, et al. LipF increases rifampicin and streptomycin sensitivity in a Mycobacterium tuberculosis surrogate. BMC Microbiol. 2020;20(1):132. doi: 10.1186/s12866-020-01802-x 32450809 PMC7249682

[6] Granados-Tristán AL, Borrego-Loya A, González-Escalante LA, Esquivel-Lucio GJ, Márquez-Uribe DY, Rigal-Leal J, et al. Role of esxG and esxH genes in drug-resistant mycobacterium. Microb Drug Resist. 2020;26(11):1279–90. doi: 10.1089/mdr.2019.0391 32379526

[7] Peñuelas-Urquides K, Bermúdez de León M, Silva-Ramírez B, Castorena-Torres F, Molina-Salinas G, del Olmo E. Two new dihydrosphingosine analogs against Mycobacterium tuberculosis affect gltA1, lprQ, and rpsO expression. Front Microbiol. 2021;12:742867. doi: 10.3389/fmicb.2021.742867 34803964 PMC8595602

[8] Tinaztepe E, Wei J-R, Raynowska J, Portal-Celhay C, Thompson V, Philips JA. Role of metal-dependent regulation of ESX-3 secretion in intracellular survival of mycobacterium tuberculosis. Infect Immun. 2016;84(8):2255–63. doi: 10.1128/IAI.00197-16 27245412 PMC4962639

[9] Siegrist MS, Unnikrishnan M, McConnell MJ, Borowsky M, Cheng T-Y, Siddiqi N, et al. Mycobacterial Esx-3 is required for mycobactin-mediated iron acquisition. Proc Natl Acad Sci U S A. 2009;106(44):18792–7. doi: 10.1073/pnas.0900589106 19846780 PMC2774023

[10] Serafini A, Pisu D, Palù G, Rodriguez GM, Manganelli R. The ESX-3 secretion system is necessary for iron and zinc homeostasis in Mycobacterium tuberculosis. PLoS One. 2013;8(10):e78351. doi: 10.1371/journal.pone.0078351 24155985 PMC3796483

[11] Tufariello JM, Chapman JR, Kerantzas CA, Wong K-W, Vilchèze C, Jones CM, et al. Separable roles for Mycobacterium tuberculosis ESX-3 effectors in iron acquisition and virulence. Proc Natl Acad Sci U S A. 2016;113(3):E348-57. doi: 10.1073/pnas.1523321113 26729876 PMC4725510

[12] Granados-Tristán AL, Hernández-Luna CE, González-Escalante LA, Camacho-Moll ME, Silva-Ramírez B, de León MB, et al. ESX-3 secretion system in Mycobacterium: An overview. Biochimie. 2023. 10.1016/j.biochi.2023.10.013 37879428

[13] Houben ENG, Bestebroer J, Ummels R, Wilson L, Piersma SR, Jiménez CR, et al. Composition of the type VII secretion system membrane complex. Mol Microbiol. 2012;86(2):472–84. doi: 10.1111/j.1365-2958.2012.08206.x 22925462

[14] Poweleit N, Czudnochowski N, Nakagawa R, Trinidad DD, Murphy KC, Sassetti CM, et al. The structure of the endogenous ESX-3 secretion system. Elife. 2019;8:e52983. doi: 10.7554/eLife.52983 31886769 PMC6986878

[15] Famelis N, Rivera-Calzada A, Degliesposti G, Wingender M, Mietrach N, Skehel JM, et al. Architecture of the mycobacterial type VII secretion system. Nature. 2019;576(7786):321–5. doi: 10.1038/s41586-019-1633-1 31597161 PMC6914368

[16] Raman K, Chandra N. Mycobacterium tuberculosis interactome analysis unravels potential pathways to drug resistance. BMC Microbiol. 2008;8:234. doi: 10.1186/1471-2180-8-234 19105810 PMC2649132

[17] Dragset MS, Poce G, Alfonso S, Padilla-Benavides T, Ioerger TR, Kaneko T, et al. A novel antimycobacterial compound acts as an intracellular iron chelator. Antimicrob Agents Chemother. 2015;59(4):2256–64. doi: 10.1128/AAC.05114-14 25645825 PMC4356758

[18] Bottai D, Serafini A, Cascioferro A, Brosch R, Manganelli R. Targeting type VII/ESX secretion systems for development of novel antimycobacterial drugs. Curr Pharm Des. 2014;20(27):4346–56. doi: 10.2174/1381612819666131118170717 24245757

[19] Wouters OJ, McKee M, Luyten J. Estimated research and development investment needed to bring a new medicine to market, 2009-2018. JAMA. 2020;323(9):844–53. doi: 10.1001/jama.2020.1166 32125404 PMC7054832

[20] Sabe VT, Ntombela T, Jhamba LA, Maguire GEM, Govender T, Naicker T, et al. Current trends in computer aided drug design and a highlight of drugs discovered via computational techniques: A review. Eur J Med Chem. 2021;224:113705. doi: 10.1016/j.ejmech.2021.113705 34303871

[21] Guedes IA, de Magalhães CS, Dardenne LE. Receptor-ligand molecular docking. Biophys Rev. 2014;6(1):75–87. doi: 10.1007/s12551-013-0130-2 28509958 PMC5425711

[22] Pushpakom S, Iorio F, Eyers PA, Escott KJ, Hopper S, Wells A, et al. Drug repurposing: progress, challenges and recommendations. Nat Rev Drug Discov. 2019;18(1):41–58. doi: 10.1038/nrd.2018.168 30310233

[23] Wang J, Brodmann M, Basler M. Assembly and subcellular localization of bacterial type VI Secretion Systems. Annu Rev Microbiol. 2019;73:621–38. doi: 10.1146/annurev-micro-020518-115420 31226022

[24] Rosell M, Fernández-Recio J. Hot-spot analysis for drug discovery targeting protein-protein interactions. Expert Opin Drug Discov. 2018;13(4):327–38. doi: 10.1080/17460441.2018.1430763 29376444

[25] Jafary F, Ganjalikhany MR, Moradi A, Hemati M, Jafari S. Novel peptide inhibitors for lactate dehydrogenase a (LDHA): a survey to inhibit LDHA activity via disruption of protein-protein interaction. Sci Rep. 2019;9(1):4686. doi: 10.1038/s41598-019-38854-7 30886157 PMC6423238

[26] Pavlinov I, Salkovski M, Aldrich LN. Beclin 1-ATG14L Protein-Protein Interaction Inhibitor Selectively Inhibits Autophagy through Disruption of VPS34 Complex I. J Am Chem Soc. 2020;142(18):8174–82. doi: 10.1021/jacs.9b12705 32320221

[27] den Blaauwen T, Andreu JM, Monasterio O. Bacterial cell division proteins as antibiotic targets. Bioorg Chem. 2014;55:27–38. doi: 10.1016/j.bioorg.2014.03.007 24755375

[28] Rock JM, Hopkins FF, Chavez A, Diallo M, Chase MR, Gerrick ER, et al. Programmable transcriptional repression in mycobacteria using an orthogonal CRISPR interference platform. Nat Microbiol. 2017;2:16274. doi: 10.1038/nmicrobiol.2016.274 28165460 PMC5302332

[29] Wang S, Zhou K, Yang X, Zhang B, Zhao Y, Xiao Y, et al. Structural insights into substrate recognition by the type VII secretion system. Protein Cell. 2020;11(2):124–37. doi: 10.1007/s13238-019-00671-z 31758528 PMC6954902

[30] Hibbs RE, Gouaux E. Principles of activation and permeation in an anion-selective Cys-loop receptor. Nature. 2011;474(7349):54–60. doi: 10.1038/nature10139 21572436 PMC3160419

[31] Riccardi L, Genna V, De Vivo M. Metal–ligand interactions in drug design. Nat Rev Chem. 2018;2(7):100–12. doi: 10.1038/s41570-018-0018-6

[32] Çınaroğlu SS, Timuçin E. Comparative assessment of seven docking programs on a nonredundant metalloprotein subset of the PDBbind Refined. J Chem Inf Model. 2019;59(9):3846–59. doi: 10.1021/acs.jcim.9b00346 31460757

[33] Carugo O, Pongor S. A normalized root-mean-square distance for comparing protein three-dimensional structures. Protein Sci. 2001;10(7):1470–3. doi: 10.1110/ps.690101 11420449 PMC2374114

[34] Cossar PJ, Lewis PJ, McCluskey A. Protein-protein interactions as antibiotic targets: a medicinal chemistry perspective. Med Res Rev. 2020;40(2):469–94. doi: 10.1002/med.21519 30004586

[35] Lu H, Zhou Q, He J, Jiang Z, Peng C, Tong R, et al. Recent advances in the development of protein-protein interactions modulators: mechanisms and clinical trials. Signal Transduct Target Ther. 2020;5(1):213. doi: 10.1038/s41392-020-00315-3 32968059 PMC7511340

[36] Moreira IS, Fernandes PA, Ramos MJ. Hot spots--a review of the protein-protein interface determinant amino-acid residues. Proteins. 2007;68(4):803–12. doi: 10.1002/prot.21396 17546660

[37] Kozakov D, Hall DR, Chuang G-Y, Cencic R, Brenke R, Grove LE, et al. Structural conservation of druggable hot spots in protein-protein interfaces. Proc Natl Acad Sci U S A. 2011;108(33):13528–33. doi: 10.1073/pnas.1101835108 21808046 PMC3158149

[38] El-Saber Batiha G, Alqahtani A, Ilesanmi OB, Saati AA, El-Mleeh A, Hetta HF, et al. Avermectin derivatives, pharmacokinetics, therapeutic and toxic dosages, mechanism of action, and their biological effects. Pharmaceuticals (Basel). 2020;13(8):196. doi: 10.3390/ph13080196 32824399 PMC7464486

[39] Tanchuk VY, Tanin VO, Vovk AI, Poda G. A new, improved hybrid scoring function for molecular docking and scoring based on autodock and autodock Vina. Chem Biol Drug Des. 2016;87(4):618–25. doi: 10.1111/cbdd.12697 26643167

[40] Lim LE, Vilchèze C, Ng C, Jacobs WR Jr, Ramón-García S, Thompson CJ. Anthelmintic avermectins kill Mycobacterium tuberculosis, including multidrug-resistant clinical strains. Antimicrob Agents Chemother. 2013;57(2):1040–6. doi: 10.1128/AAC.01696-12 23165468 PMC3553693

[41] Muhammed Ameen S, Drancourt M. Ivermectin lacks antituberculous activity. J Antimicrob Chemother. 2013;68(8):1936–7. doi: 10.1093/jac/dkt089 23587653

[42] Ramón-García S, Vilchèze C, Lim LE, Ng C, Jacobs WR Jr, Thompson CJ. Measurements of the in vitro anti-mycobacterial activity of ivermectin are method-dependent. J Antimicrob Chemother. 2014;69(6):1723–4. doi: 10.1093/jac/dku037 24569629

[43] Filimonov DA, Lagunin AA, Gloriozova TA, Rudik AV, Druzhilovskii DS, Pogodin PV, et al. Prediction of the biological activity spectra of organic compounds using the pass online web resource. Chem Heterocycl Comp. 2014;50(3):444–57. doi: 10.1007/s10593-014-1496-1

[44] Daina A, Michielin O, Zoete V. SwissADME: a free web tool to evaluate pharmacokinetics, drug-likeness and medicinal chemistry friendliness of small molecules. Sci Rep. 2017;7:42717. doi: 10.1038/srep42717 28256516 PMC5335600

[45] Banerjee P, Eckert AO, Schrey AK, Preissner R. ProTox-II: a webserver for the prediction of toxicity of chemicals. Nucleic Acids Res. 2018;46(W1):W257–63. doi: 10.1093/nar/gky318 29718510 PMC6031011

[46] Omansen TF, Porter JL, Johnson PDR, van der Werf TS, Stienstra Y, Stinear TP. In-vitro activity of avermectins against Mycobacterium ulcerans. PLoS Negl Trop Dis. 2015;9(3):e0003549. doi: 10.1371/journal.pntd.0003549 25742173 PMC4351077

[47] Fukano H, Ikeda A, Hirose T, Iwatsuki M, Hasegawa N, Sunazuka T, . In vitro/ex vivo activity of avermectin derivatives against clarithromycin resistant Mycobacterium avium. Am Thoracic Soc Int Conference Abstracts. 2021.

[48] Varela C, Rittmann D, Singh A, Krumbach K, Bhatt K, Eggeling L, et al. MmpL genes are associated with mycolic acid metabolism in mycobacteria and corynebacteria. Chem Biol. 2012;19(4):498–506. doi: 10.1016/j.chembiol.2012.03.006 22520756 PMC3370651

[49] Siegrist MS, Steigedal M, Ahmad R, Mehra A, Dragset MS, Schuster BM, et al. Mycobacterial Esx-3 requires multiple components for iron acquisition. MBio. 2014;5(3):e01073-14. doi: 10.1128/mBio.01073-14 24803520 PMC4010830

[50] Nath Y, Ray SK, Buragohain AK. Essential role of the ESX-3 associated eccD3 locus in maintaining the cell wall integrity of Mycobacterium smegmatis. Int J Med Microbiol. 2018;308(7):784–95. doi: 10.1016/j.ijmm.2018.06.010 30257807

[51] Xiao J, Jia H, Pan L, Li Z, Lv L, Du B, et al. Application of the CRISPRi system to repress sepF expression in Mycobacterium smegmatis. Infect Genet Evol. 2019;72:183–90. doi: 10.1016/j.meegid.2018.06.033 31242975

[52] Faulkner V, Cox AA, Goh S, van Bohemen A, Gibson AJ, Liebster O, et al. Re-sensitization of mycobacterium smegmatis to rifampicin using CRISPR interference demonstrates its utility for the study of non-essential drug resistance traits. Front Microbiol. 2021;11:619427. doi: 10.3389/fmicb.2020.619427 33597931 PMC7882622

[53] Woerde DJ, Martin PA, Govendir M. Susceptibility of rapidly growing mycobacteria isolated from Australian cats to ivermectin, moxidectin, ceftiofur and florfenicol. J Feline Med Surg. 2015;17(12):1065–8. doi: 10.1177/1098612X14565497 25572306 PMC10816349

[54] Depardieu F, Podglajen I, Leclercq R, Collatz E, Courvalin P. Modes and modulations of antibiotic resistance gene expression. Clin Microbiol Rev. 2007;20(1):79–114. doi: 10.1128/CMR.00015-06 17223624 PMC1797629

[55] Piddington DL, Kashkouli A, Buchmeier NA. Growth of Mycobacterium tuberculosis in a defined medium is very restricted by acid pH and Mg(2+) levels. Infect Immun. 2000;68(8):4518–22. doi: 10.1128/IAI.68.8.4518-4522.2000 10899850 PMC98362

[56] Voskuil MI, Bartek IL, Visconti K, Schoolnik GK. The response of mycobacterium tuberculosis to reactive oxygen and nitrogen species. Front Microbiol. 2011;2:105. doi: 10.3389/fmicb.2011.00105 21734908 PMC3119406

[57] Wang L, Asare E, Shetty AC, Sanchez-Tumbaco F, Edwards MR, Saranathan R, et al. Multiple genetic paths including massive gene amplification allow Mycobacterium tuberculosis to overcome loss of ESX-3 secretion system substrates. Proc Natl Acad Sci U S A. 2022;119(8):e2112608119. doi: 10.1073/pnas.2112608119 35193958 PMC8872769

[58] Waterhouse A, Bertoni M, Bienert S, Studer G, Tauriello G, Gumienny R, et al. SWISS-MODEL: homology modelling of protein structures and complexes. Nucleic Acids Res. 2018;46(W1):W296–303. doi: 10.1093/nar/gky427 29788355 PMC6030848

[59] Pettersen EF, Goddard TD, Huang CC, Couch GS, Greenblatt DM, Meng EC, et al. UCSF Chimera--a visualization system for exploratory research and analysis. J Comput Chem. 2004;25(13):1605–12. doi: 10.1002/jcc.20084 15264254

[60] Williams CJ, Headd JJ, Moriarty NW, Prisant MG, Videau LL, Deis LN, et al. MolProbity: more and better reference data for improved all-atom structure validation. Protein Sci. 2018;27(1):293–315. doi: 10.1002/pro.3330 29067766 PMC5734394

[61] Mourenza Á, Gil JA, Mateos LM, Letek M. Novel treatments against mycobacterium tuberculosis based on drug repurposing. Antibiotics (Basel). 2020;9(9):550. doi: 10.3390/antibiotics9090550 32872158 PMC7557778

[62] Kawabata T. Detection of cave pockets in large molecules: Spaces into which internal probes can enter, but external probes from outside cannot. Biophys Physicobiol. 2019;16:391–406. doi: 10.2142/biophysico.16.0_391 31984193 PMC6975925

[63] Kim S, Chen J, Cheng T, Gindulyte A, He J, He S, et al. PubChem in 2021: new data content and improved web interfaces. Nucleic Acids Res. 2021;49(D1):D1388–95. doi: 10.1093/nar/gkaa971 33151290 PMC7778930

[64] Morris GM, Huey R, Lindstrom W, Sanner MF, Belew RK, Goodsell DS, et al. AutoDock4 and AutoDockTools4: automated docking with selective receptor flexibility. J Comput Chem. 2009;30(16):2785–91. doi: 10.1002/jcc.21256 19399780 PMC2760638

[65] Krissinel E, Henrick K. Inference of macromolecular assemblies from crystalline state. J Mol Biol. 2007;372(3):774–97. doi: 10.1016/j.jmb.2007.05.022 17681537

[66] Trott O, Olson AJ. AutoDock Vina: improving the speed and accuracy of docking with a new scoring function, efficient optimization, and multithreading. J Comput Chem. 2010;31(2):455–61. doi: 10.1002/jcc.21334 19499576 PMC3041641

[67] Wallace AC, Laskowski RA, Thornton JM. LIGPLOT: a program to generate schematic diagrams of protein-ligand interactions. Protein Eng. 1995;8(2):127–34. doi: 10.1093/protein/8.2.127 7630882

[68] Pettersen EF, Goddard TD, Huang CC, Meng EC, Couch GS, Croll TI, et al. UCSF ChimeraX: structure visualization for researchers, educators, and developers. Protein Sci. 2021;30(1):70–82. doi: 10.1002/pro.3943 32881101 PMC7737788

[69] Schmittgen TD, Livak KJ. Analyzing real-time PCR data by the comparative C(T) method. Nat Protoc. 2008;3(6):1101–8. doi: 10.1038/nprot.2008.73 18546601

[70] King A, Eckersly R. Inferential statistics IV: choosing a hypothesis test. Statist Biomed Eng Scient. 2019:147–71.

[71] Pandey M, Talwar S, Pal R, Nain V, Johri S, Singhal A, et al. Transcription factor mce3R modulates antibiotics and disease persistence in Mycobacteriumtuberculosis. Res Microbiol. 2023;174(7):104082. doi: 10.1016/j.resmic.2023.104082 37244349

[72] Mohapatra S, Rajesh N, Rajesh V. Impact of heavy metal lead stress on polyamine levels in Halomonas BVR 1 isolated from an industry effluent. Sci Rep. 2017;7(1):13447. doi: 10.1038/s41598-017-13893-0 29044167 PMC5647450

