# Supplementary material for: Mycobacterium susceptibility to ivermectin by inhibition of eccD3, an ESX-3 secretion system component
Source: PLoS Comput Biol. 2025 Apr 17;21(4):e1012936. doi: 10.1371/journal.pcbi.1012936 (PMC12005495; doi:10.1371/journal.pcbi.1012936)
Supplement: S1 Fig — Experimental pose (red) and the predicted molecular docking pose (green). EccC3 ATPase domain III experimental data 1-PDB ID 6J17 [29]. (DOCX) [file pcbi.1012936.s001.docx]

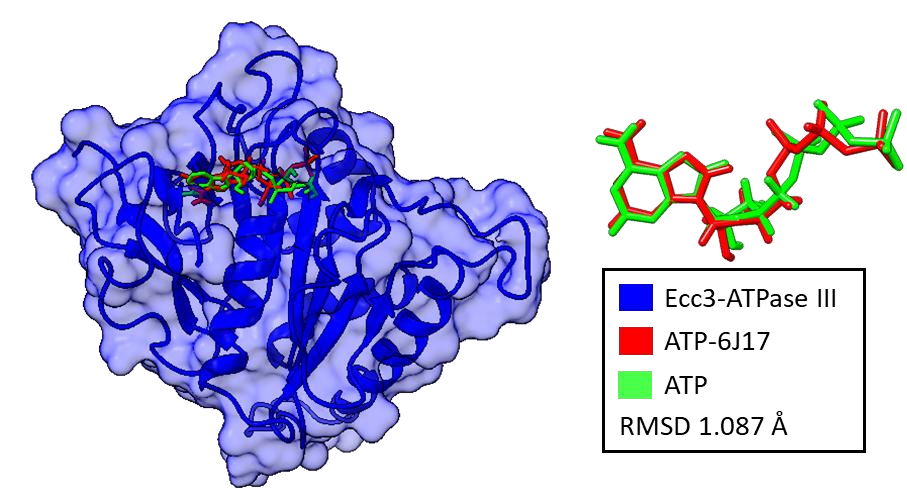


S1 Fig. EccC3 ATPase domain III structural superposition of the ATP. Experimental pose (red) and the predicted molecular docking pose (green). EccC3 ATPase domain III experimental data 1-PDB ID 6J17 ^29^.
