# Supplementary material for: Mycobacterium susceptibility to ivermectin by inhibition of eccD3, an ESX-3 secretion system component
Source: PLoS Comput Biol. 2025 Apr 17;21(4):e1012936. doi: 10.1371/journal.pcbi.1012936 (PMC12005495; doi:10.1371/journal.pcbi.1012936)
Supplement: S2 Fig — Protomer 2 interactions with the three drugs having Z-score values less than -1. EccD3-1, refers to extended monomer and EccD3-2, refers to bent monomer. (DOCX) [file pcbi.1012936.s002.docx]

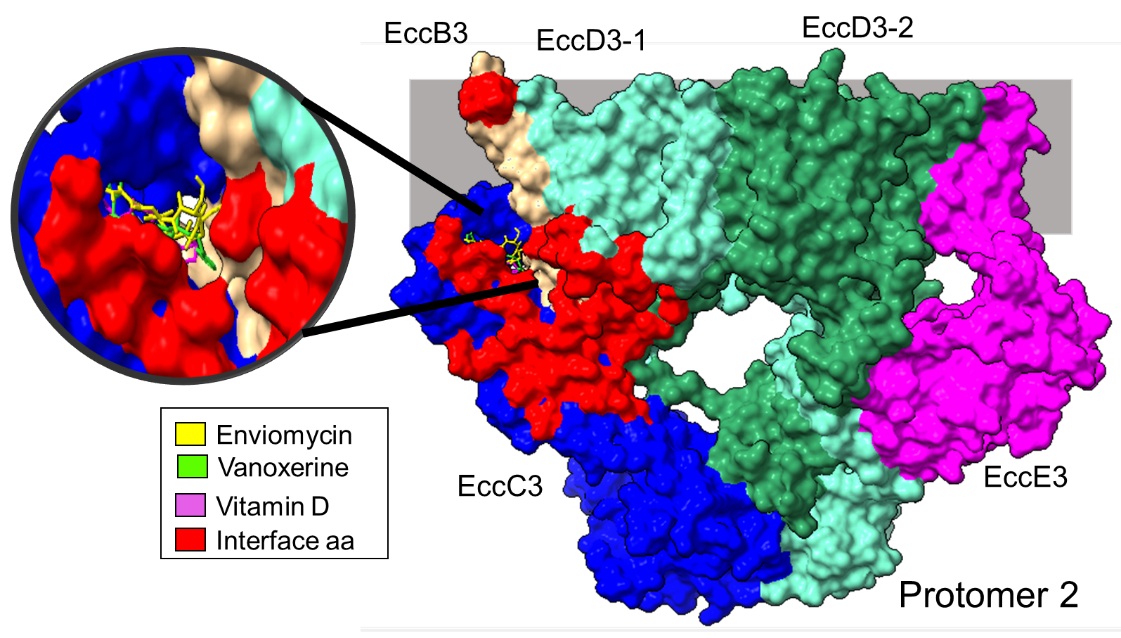


S2 Fig. *M. tuberculosis* ESX-3 secretion system protomer 2. Protomer 2 interactions with the three drugs having Z-score values less than -1. EccD3-1, refers to extended monomer and EccD3-2, refers to bent monomer.
