# Supplementary material for: Mycobacterium susceptibility to ivermectin by inhibition of eccD3, an ESX-3 secretion system component
Source: PLoS Comput Biol. 2025 Apr 17;21(4):e1012936. doi: 10.1371/journal.pcbi.1012936 (PMC12005495; doi:10.1371/journal.pcbi.1012936)
Supplement: S3 Fig — (a) Docking between C chain and D chain glutamate-gated chloride channel of C. elegans with ivermectin. Structural superposition of ivermectin-experimental pose (red, 2-PDB ID 3RIF) and ivermectin-docking pose [30]. Molecular interactions in 2D diagram of glutamate-gated chloride channel and ivermectin (b) experimental data, and (c) molecular docking data. The 2D diagrams (c) and (d) represent amino acids of the corresponding glutamate-gated chloride channel C and D chains, respectively. Ligplot was used for 2D map. (DOCX) [file pcbi.1012936.s003.docx]

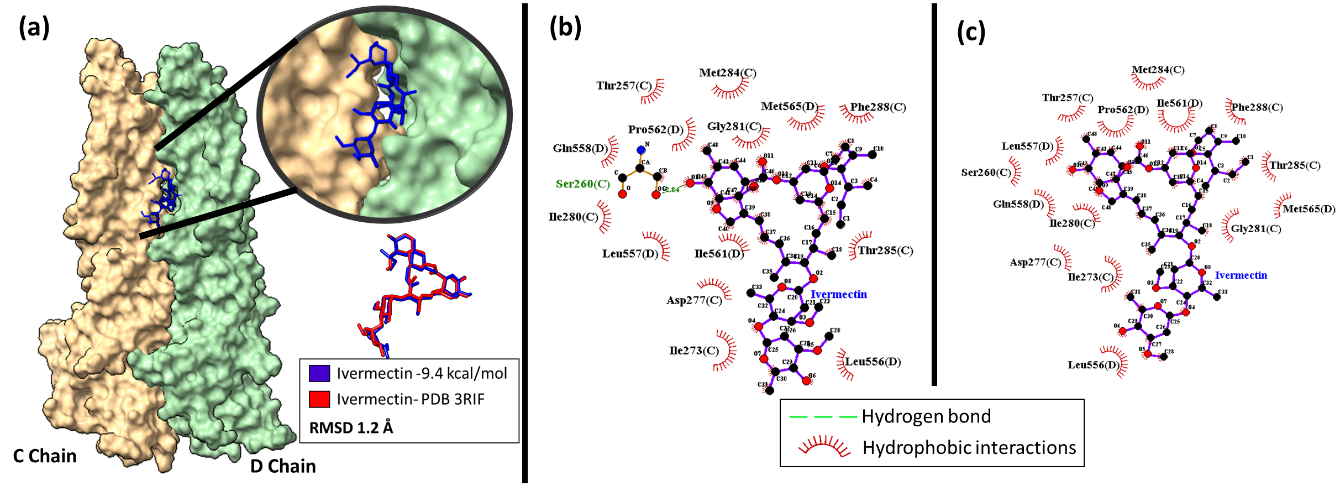
S3 Fig. Glutamate-gated chloride-channel of *C. elegans* and ivermectin molecular docking. (a) Docking between C chain and D chain glutamate-gated chloride channel of *C. elegans* with ivermectin. Structural superposition of ivermectin-experimental pose (red, 2-PDB ID 3RIF) and ivermectin-docking pose ^30^. Molecular interactions in 2D diagram of glutamate-gated chloride channel and ivermectin (b) experimental data, and (c) molecular docking data. The 2D diagrams (c) and (d) represent amino acids of the corresponding glutamate-gated chloride channel C and D chains, respectively. Ligplot was used for 2D map.
