# Supplementary material for: Mycobacterium susceptibility to ivermectin by inhibition of eccD3, an ESX-3 secretion system component
Source: PLoS Comput Biol. 2025 Apr 17;21(4):e1012936. doi: 10.1371/journal.pcbi.1012936 (PMC12005495; doi:10.1371/journal.pcbi.1012936)
Supplement: S4 Fig — Bars represent the frequency of each type of amino acids of each drug-target. Interface amino acids interactions with selamectin, moxidectin and rifalazil are illustrated. (DOCX) [file pcbi.1012936.s004.docx]

S4 Fig. Interface amino acids of the interaction protein-drug. Bars represent the frequency of each type of amino acids of each drug-target. Interface amino acids interactions with selamectin, moxidectin and rifalazil are illustrated.
