# Supplementary material for: Mycobacterium susceptibility to ivermectin by inhibition of eccD3, an ESX-3 secretion system component
Source: PLoS Comput Biol. 2025 Apr 17;21(4):e1012936. doi: 10.1371/journal.pcbi.1012936 (PMC12005495; doi:10.1371/journal.pcbi.1012936)
Supplement: S5 Fig — The hydrophobicity of EccD3 (a) and EccE3 (b) structures hydrophilicity is represented in blue while hydrophobicity in red. Drug binding site of EccD3 (c) and EccE3 (d) is shown in red color. (DOCX) [file pcbi.1012936.s005.docx]

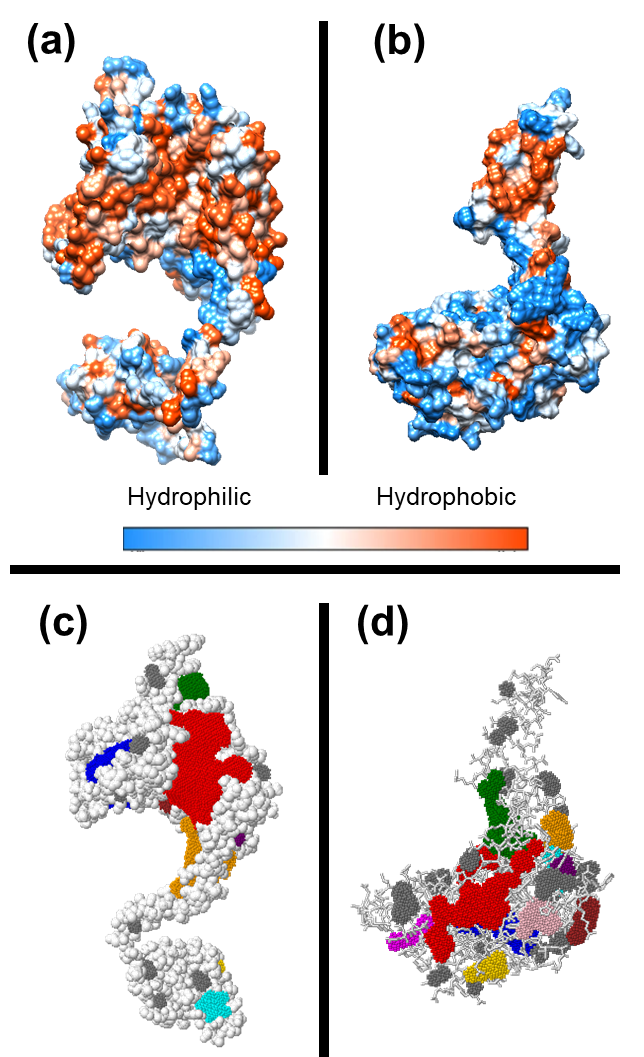


S5 Fig. EccD3 and EccE3 3D structures. The hydrophobicity of EccD3 (a) and EccE3 (b) structures hydrophilicity is represented in blue while hydrophobicity in red. Drug binding site of EccD3 (c) and EccE3 (d) is shown in red color.
