# Supplementary material for: Mycobacterium susceptibility to ivermectin by inhibition of eccD3, an ESX-3 secretion system component
Source: PLoS Comput Biol. 2025 Apr 17;21(4):e1012936. doi: 10.1371/journal.pcbi.1012936 (PMC12005495; doi:10.1371/journal.pcbi.1012936)
Supplement: S6 Fig — (a) EccC3 ATPase domain III interactions with the three drugs having Z-scores values less than -1 and the ATP molecule as control. Molecular interactions between the EccC3 ATPase domain III interface amino acids with (b) moxidectin, (c) enviomycin, and (d) levofloxacin in 2D diagram. Ligplot was used for 2D map. (DOCX) [file pcbi.1012936.s006.docx]

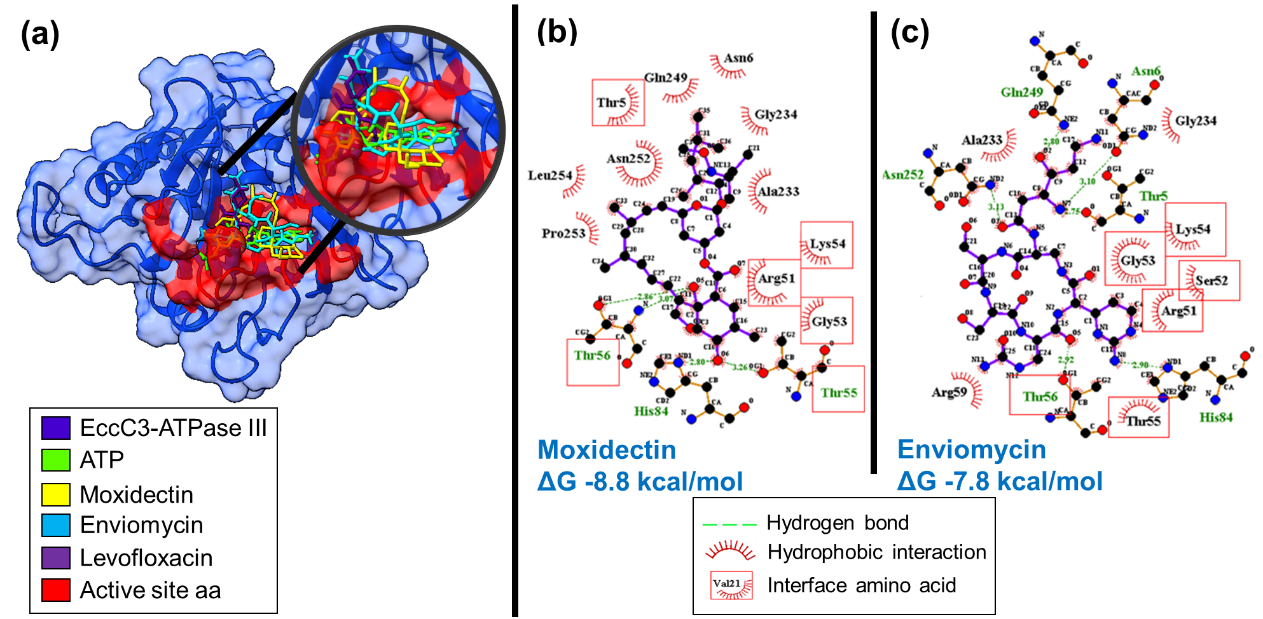


S6 Fig. *M. tuberculosis* ESX-3 EccC3 ATPase domain III. (a) EccC3 ATPase domain III interactions with the three drugs having Z-scores values less than -1 and the ATP molecule as control. Molecular interactions between the EccC3 ATPase domain III interface amino acids with (b) moxidectin, (c) enviomycin, and (d) levofloxacin in 2D diagram. Ligplot was used for 2D map.
