# Supplementary material for: Mycobacterium susceptibility to ivermectin by inhibition of eccD3, an ESX-3 secretion system component
Source: PLoS Comput Biol. 2025 Apr 17;21(4):e1012936. doi: 10.1371/journal.pcbi.1012936 (PMC12005495; doi:10.1371/journal.pcbi.1012936)
Supplement: S7 Fig — Resazurin Microtiter Assay plate method with serial dilutions of ivermectin 4μM to 0.03 μM (4096 μg/mL to 8 μg/mL), MIC was determined at 128 μg/mL. Each experiment was performed in technical triplicates shown in rows. (DOCX) [file pcbi.1012936.s007.docx]

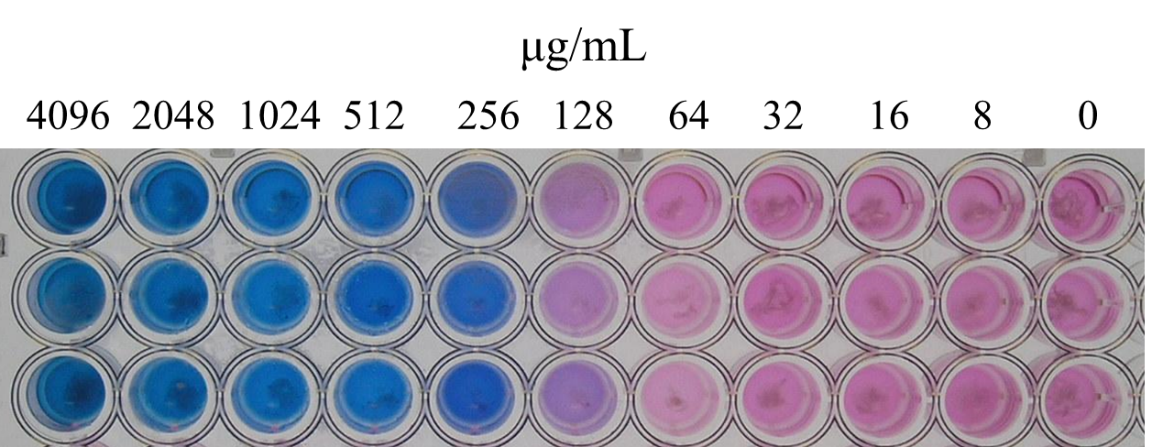


S7 Fig. Ivermectin minimum inhibitory concentration (MIC) determination of *M. smegmatis* wild type strain. Resazurin Microtiter Assay plate method with serial dilutions of ivermectin 4μM to 0.03 μM (4096 μg/mL to 8 μg/mL), MIC was determined at 128 μg/mL. Each experiment was performed in technical triplicates shown in rows.
