# Supplementary material for: Mycobacterium susceptibility to ivermectin by inhibition of eccD3, an ESX-3 secretion system component
Source: PLoS Comput Biol. 2025 Apr 17;21(4):e1012936. doi: 10.1371/journal.pcbi.1012936 (PMC12005495; doi:10.1371/journal.pcbi.1012936)
Supplement: S8 Fig — Resazurin Microtiter Assay plate method with serial dilutions of ivermectin from 0.5 μM to 0.0009 μM (512 μg/mL to 1 μg/mL). Ivermectin MIC of M. smegmatis PLJR962-eccD3-gRNA strain without (a) or with ATc (anhydrotetracycline 0.0002 μM (100 ng/mL) (b) was determined at 128 μg/mL and 64 μg/mL respectively, and MIC of M. smegmatis PLJR962-control-gRNA strain without (c) or with ATc 0.0002 μM (100 ng/mL) (d) were observed at 128 μg/mL. Each experiment was performed in technical triplicates shown in rows. (DOCX) [file pcbi.1012936.s008.docx]

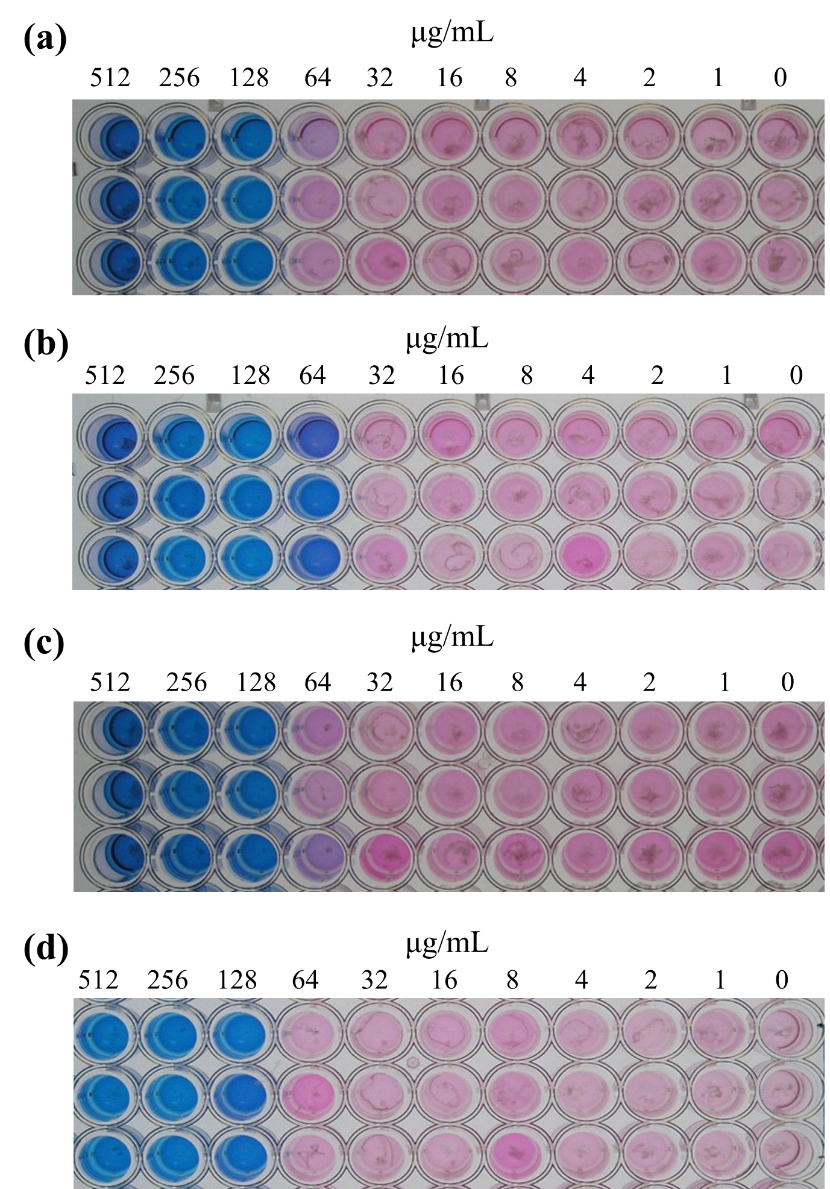


S8 Fig. Ivermectin minimum inhibitory concentration (MIC) determination of *M. smegmatis* PLJR962-*eccD3*-gRNA and *M. smegmatis* PLJR962-control*-*gRNA strains. Resazurin Microtiter Assay plate method with serial dilutions of ivermectin from 0.5 μM to 0.0009 μM (512 μg/mL to 1 μg/mL). Ivermectin MIC of *M. smegmatis* PLJR962-*eccD3*-gRNA strain without (a) or with ATc (anhydrotetracycline 0.0002 μM (100 ng/mL) (b) was determined at 128 μg/mL and 64 μg/mL respectively, and MIC of *M. smegmatis* PLJR962-control*-*gRNA strain without (c) or with ATc 0.0002 μM (100 ng/mL) (d) were observed at 128 μg/mL. Each experiment was performed in technical triplicates shown in rows.
