# Supplementary material for: Mycobacterium susceptibility to ivermectin by inhibition of eccD3, an ESX-3 secretion system component
Source: PLoS Comput Biol. 2025 Apr 17;21(4):e1012936. doi: 10.1371/journal.pcbi.1012936 (PMC12005495; doi:10.1371/journal.pcbi.1012936)
Supplement: S9 Fig — Graph of relative values of absorbance measured at 605 nm for (a) M. smegmatis PLJR962-eccD3-gRNA strain and (b) M. smegmatis PLJR962-control-gRNA strain without or with ATc 0.0002 μM (100 ng/mL). A low level of absorbance corresponds to pink well, whereas high absorbance is for blue wells. IVM (ivermectin) 0.0009 μM to 0.5 μM. Using Synergy HT (BioTek) microplate reader. Each experiment was performed in technical triplicates showing the average and error bars for standard deviation. (DOCX) [file pcbi.1012936.s009.docx]

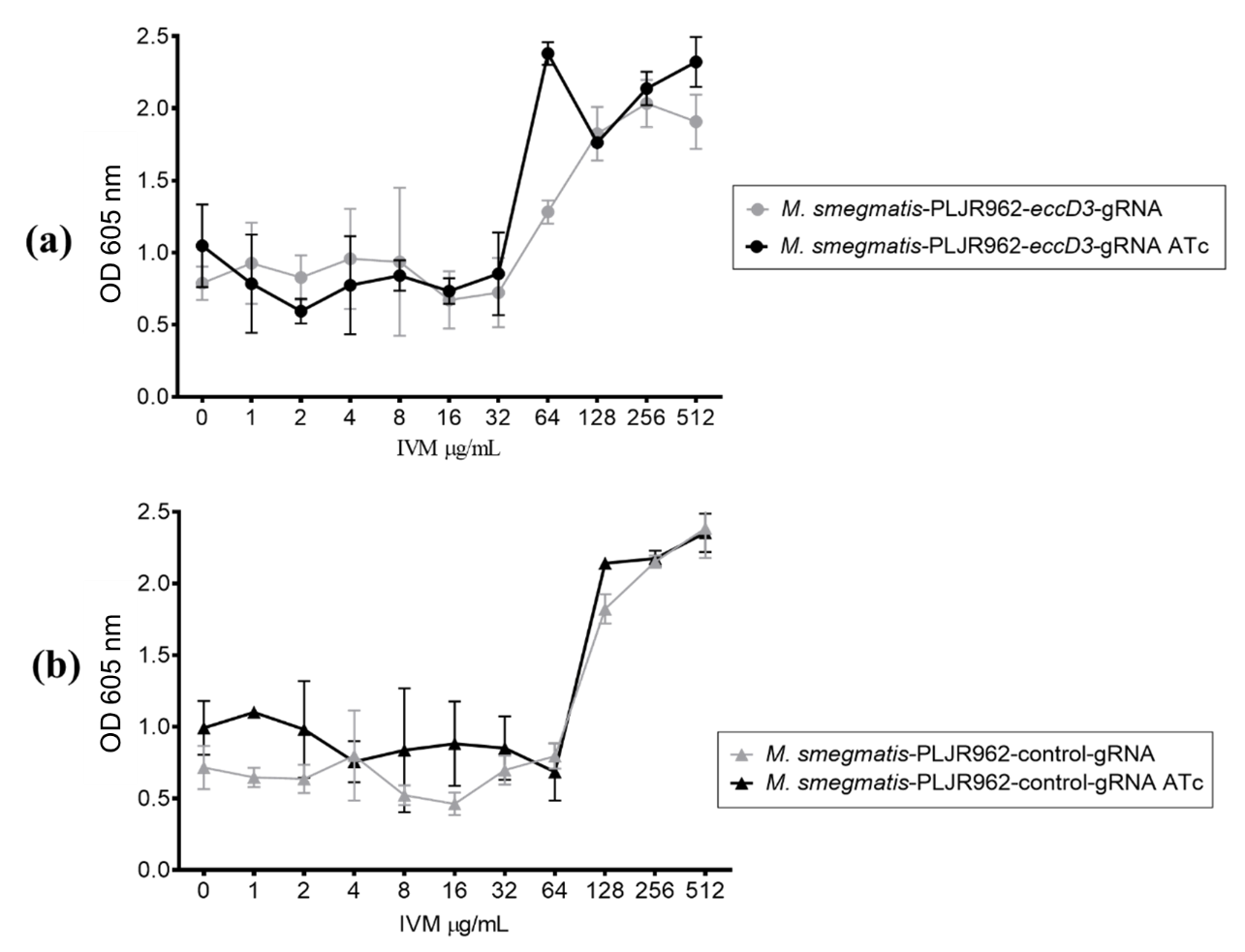


S9 Fig. Ivermectin MIC of *M. smegmatis* PLJR962-*eccD3*-gRNA and *M. smegmatis* PLJR962-control*-*gRNA strains. Graph of relative values of absorbance measured at 605 nm for (a) *M. smegmatis* PLJR962-*eccD3*-gRNA strain and (b) *M. smegmatis* PLJR962-control*-*gRNA strain without or with ATc 0.0002 μM (100 ng/mL). A low level of absorbance corresponds to pink well, whereas high absorbance is for blue wells. IVM (ivermectin) 0.0009 μM to 0.5 μM. Using Synergy HT (BioTek) microplate reader. Each experiment was performed in technical triplicates showing the average and error bars for standard deviation
