# Supplementary material for: Mycobacterium susceptibility to ivermectin by inhibition of eccD3, an ESX-3 secretion system component
Source: PLoS Comput Biol. 2025 Apr 17;21(4):e1012936. doi: 10.1371/journal.pcbi.1012936 (PMC12005495; doi:10.1371/journal.pcbi.1012936)
Supplement: S10 Fig — Growth curve of M. smegmatis PLJR962-eccD3-gRNA strain (circles) and M. smegmatis PLJR962-control-gRNA strain (triangles) without (gray line) and with (black line) ATc 0.0002 μM (100 ng/mL), with a final concentration of (A) 0.5 μM (512 μg/mL), (B) 0.25 μM (256 μg/mL), and (C) 0.125 μM (128 μg/mL) of ivermectin, and 0.03 μM (20 μg/mL) of kanamycin. Each experiment was performed in technical triplicates showing the average and respective error bars for standard deviation. (DOCX) [file pcbi.1012936.s010.docx]

S10 Fig. Growth curve of *M. smegmatis* PLJR962-*eccD3*-gRNA strain with ivermectin. Growth curve of *M. smegmatis* PLJR962-*eccD3*-gRNA strain (circles) and *M. smegmatis* PLJR962-control*-*gRNA strain (triangles) without (gray line) and with (black line) ATc 0.0002 μM (100 ng/mL), with a final concentration of (A) 0.5 μM (512 μg/mL), (B) 0.25 μM (256 μg/mL), and (C) 0.125 μM (128 μg/mL) of ivermectin, and 0.03 μM (20 μg/mL) of kanamycin. Each experiment was performed in technical triplicates showing the average and respective error bars for standard deviation.
