# Supplementary material for: Mycobacterium susceptibility to ivermectin by inhibition of eccD3, an ESX-3 secretion system component
Source: PLoS Comput Biol. 2025 Apr 17;21(4):e1012936. doi: 10.1371/journal.pcbi.1012936 (PMC12005495; doi:10.1371/journal.pcbi.1012936)
Supplement: S12 Fig — Each gblock sequence has a marker above. Random sequences of 5’ and 3’ regions are underlined. Primer sequences and enzyme restrciction sequences are indicated with the corresponce name. The start and the end of the 247 bp PLJR962 plasmid sequence (eccD3-gRNA included) are indicated with two slash symbols (//). eccD3-gRNA sequence is indicated with bold letters and gray color. Asterisk symbols indicate the restriction enzymes (EcoRI and SalI) using to clone eccD3-gRNA fragment in PLJR962 plasmid. (DOCX) [file pcbi.1012936.s012.docx]

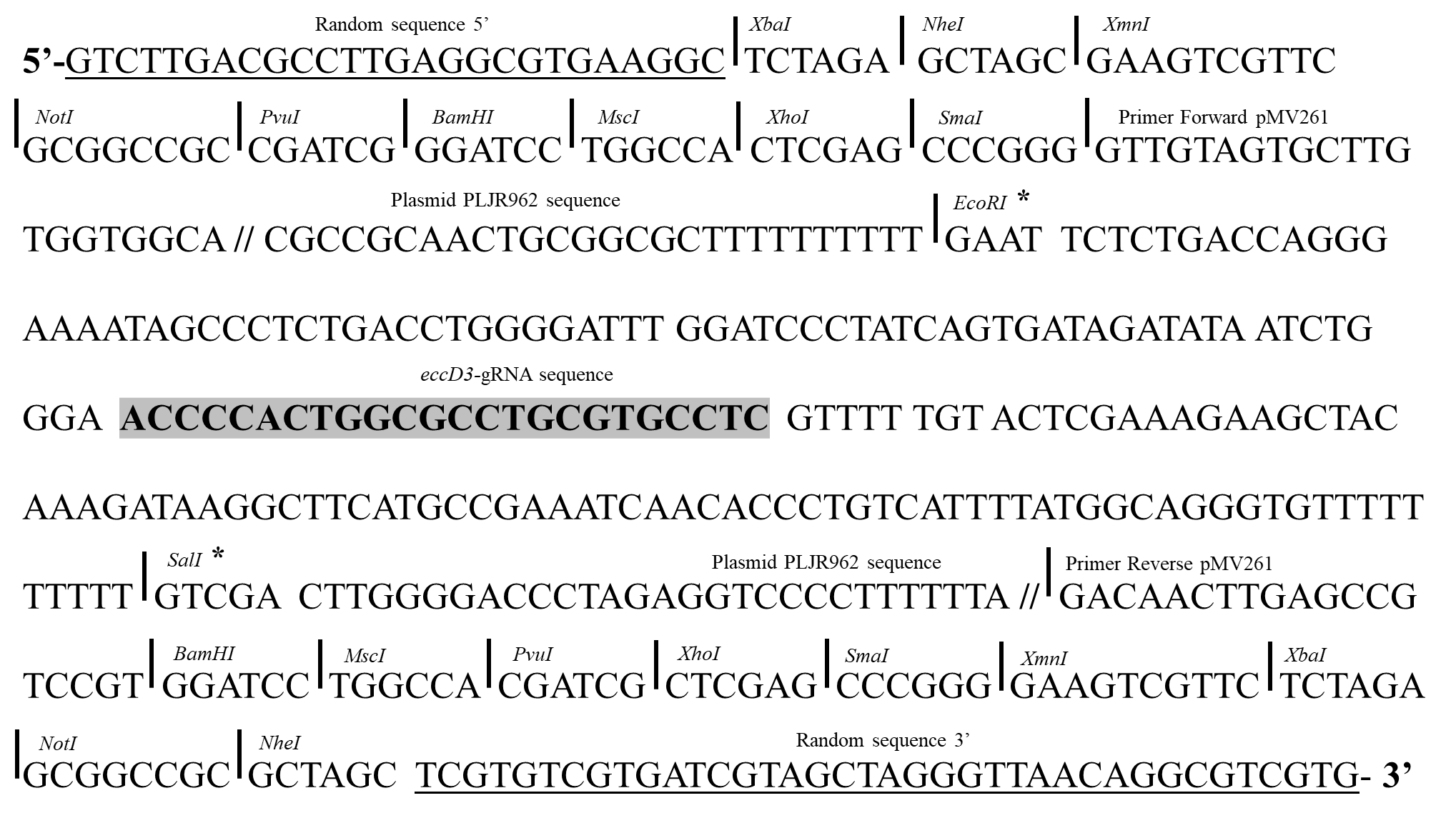


S12 Fig. Features of the gblock sequence to *eccD3*-gRNA cloning. Each gblock sequence has a marker above. Random sequences of 5’ and 3’ regions are underlined. Primer sequences and enzyme restrciction sequences are indicated with the corresponce name. The start and the end of the 247 bp PLJR962 plasmid sequence (*eccD3*-gRNA included) are indicated with two slash symbols (//). *eccD3*-gRNA sequence is indicated with bold letters and gray color. Asterisk symbols indicate the restriction enzymes (*EcoRI* and *SalI*) using to clone *eccD3*-gRNA fragment in PLJR962 plasmid.
