# Supplementary material for: Mycobacterium susceptibility to ivermectin by inhibition of eccD3, an ESX-3 secretion system component
Source: PLoS Comput Biol. 2025 Apr 17;21(4):e1012936. doi: 10.1371/journal.pcbi.1012936 (PMC12005495; doi:10.1371/journal.pcbi.1012936)
Supplement: S1 Table — (DOCX) [file pcbi.1012936.s013.docx]

S1 Table. Structure models of *M. tuberculosis* quality.

|  | **Protein** | **Mol Probity Score** | **Clash Score** | **Ramachandran outliers (%)** | **Ramachandran favored (%)** |
| --- | --- | --- | --- | --- | --- |
| ESX-3 protomer 1 | EccB3 transmembrane domain | 1.00 | 0.75 | 0 | 96.20 |
|  | EccB3 periplasm domain | 1.97 | 2.02 | 1.46 | 89.78 |
|  | EccC3 stalk and DUF domains | 1.93 | 5.89 | 3.75 | 87.75 |
|  | EccD3 bent monomer | 1.77 | 6.64 | 1.94 | 93.98 |
|  | EccD3 extended monomer | 1.21 | 1.88 | 0.65 | 96.13 |
|  | EccE3 | 1.87 | 9.31 | 1.38 | 94.48 |
| ESX-3 protomer 2 | EccB3 transmembrane domain | 0.72 | 0 | 0 | 98.31 |
|  | EccB3 periplasm domain | 1.97 | 2.02 | 1.46 | 89.78 |
|  | EccC3 stalk and DUF domains | 1.90 | 4.77 | 2.75 | 88.50 |
|  | EccD3 bent monomer | 1.82 | 6.64 | 2.15 | 92.90 |
|  | EccD3 extended monomer | 1.61 | 3.32 | 0.86 | 94.84 |
|  | EccE3 | 1.15 | 0.66 | 2.07 | 95.17 |
| Ramachandran outliers and favored refer to those amino acids with non-favorable and favorable dihedral angles, respectively. Structure quality was obtained by MolProbity server. | | | | | |
