# Supplementary material for: Mycobacterium susceptibility to ivermectin by inhibition of eccD3, an ESX-3 secretion system component
Source: PLoS Comput Biol. 2025 Apr 17;21(4):e1012936. doi: 10.1371/journal.pcbi.1012936 (PMC12005495; doi:10.1371/journal.pcbi.1012936)
Supplement: S2 Table — (DOCX) [file pcbi.1012936.s014.docx]

S2 Table. Binding sites of possible drug interaction in ESX-3 secretion system components.

| **ESX-3 component** | **Amino acids potentially recognized** |
| --- | --- |
| EccB3 | ARG 110, SER 111, THR 112, ALA 113, ALA 114, LEU 115, LEU 127, ASN 128, LEU 129, ALA 132, ARG 133, SER 141, PRO 142, THR 144, LEU 158, PRO 162, GLY 163, GLU 166, ARG 167, THR 417, VAL 418, GLU 419, LEU 420, GLY 424, ASN 425, ALA 435, ALA 436, ASP 462, THR 463, VAL 504, PRO 505, GLY 506, PRO 507, LEU 516. |
| EccD3 N-terminal | ILE 123, HIS 125, ILE 126, GLN 127, GLY 129, ALA 130, ALA 133, VAL 134, ALA 136, VAL 137, LEU 140, ALA 141, LEU 144, THR 145, THR 147, TYR 148, ALA 151, THR 152, ALA 160, ILE 164, ARG 178. |
| EccD3 C-terminal | ASP 375, ALA 377, LYS 380, ALA 381, LEU 384, TYR 388, GLY 392, LEU 395, VAL 396, THR 399, TYR 404, VAL 405, ALA 407, PHE 408, VAL 411, LEU 414, ALA 415, MET 418, TRP 421, VAL 422, VAL 424, ALA 425, LEU 426, PRO 428, ALA 431. |
| ESX-3 protomer 1 | **EccB3**- PHE 1, ALA 2, SER 3, ARG 4, THR 5, PRO 6, VAL 7, ASN 8, ASN 9, ASN 10, PRO 11, ASP 12, LYS 13, VAL 14, TYR 16, ARG 17, ARG 18, GLY 19, PHE 20, VAL 21, THR 22, ARG 23, GLN 25, VAL 26, TRP 29, ARG 30, LEU 78, ILE 79. **EccC3**- LEU 111, ARG 113, VAL 114, ILE 115, PRO 116, PRO 117, SER 118, LEU 119, ARG 122, LEU 124, ARG 167, ASN 169, MET 173, ARG 174, GLU 260, ASP 262. **EccD3-extended monomer**- LEU 1233, LEU 1239, PRO 1240, ILE 1242, ALA 1244, PRO 1245, GLY 1246, ASP 1247. |
| ESX-3 protomer 2 | **EccB3**- LEU 22, HIS 23, ASP 24, THR 25, ARG 26, LEU 28, VAL 29, PRO 31, ARG 37, PHE 56, ILE 59, ARG 60. **EccC3**- ILE 84, GLU 86, ALA 87, PRO 88, PRO 89, GLU 90, ARG 93, ARG 101, ARG 102, MET 153, ARG 154, GLU 156, GLU 157, ALA 160, GLU 161, ASP 164, TYR 165, ARG 167, TYR 168, SER 170, VAL 171, VAL 172, ASP 174, ASN 175, ILE 176, ARG 177, ALA 178, ALA 181. **EccD3-bent monomer**- ASP 566, MET 569, ILE 570, THR 573, PRO 578, 579 TRP, GLY 580. **EccD3-extended monomer**- ARG 1297, VAL 1298, TRP 1299, ASP 1300, LYS 1305, ALA 1356, SER 1357, PRO 1358, GLU 1359, SER 1360, TYR 1361, SER 1362, LEU 1363, PRO 1364, ARG 1366, ARG 1367, LEU 1368, LEU 1371. |
| EccC3 ATPase III domain | THR 1064, ALA 1109, ARG 1110, SER 1111, LYS 1113, THR 1114, THR 1115, ARG 1141, ARG 1245, ALA 1292, LEU 1309, ASN 1311. |
| Amino acids are represented by three letter code and their position is regards to protein sequence. | |
