# Supplementary material for: Mycobacterium susceptibility to ivermectin by inhibition of eccD3, an ESX-3 secretion system component
Source: PLoS Comput Biol. 2025 Apr 17;21(4):e1012936. doi: 10.1371/journal.pcbi.1012936 (PMC12005495; doi:10.1371/journal.pcbi.1012936)
Supplement: S3 Table — (DOCX) [file pcbi.1012936.s015.docx]

S3 Table. EccC3 ATPase domain III amino acids of the active site bound to ATP.

| **Active site amino acids interacted with ATP** | | |
| --- | --- | --- |
| **ATP/Mg 1-PDB ID 6J17** | **ATP -8.8 kcal/mol *** | **ATP/Mg -10.4 kcal/mol *** |
| THR 5 | THR 5 | THR 5 |
| ALA 50 | ALA 50 | ALA 50 |
| ARG 51 | ARG 51 | ARG 51 |
| SER 52 | -- | SER 52 |
| GLY 53 | GLY 53 | GLY 53 |
| LYS 54 | LYS 54 | LYS 54 |
| THR 55 | THR 55 | THR 55 |
| THR 56 | THR 56 | THR 56 |
| ARG 82 | ARG 82 | ARG 82 |
| -- | ARG 186 | ARG 186 |
| ALA 233 | ALA 233 | ALA 233 |
| GLY 234 | GLY 234 | GLY 234 |
| -- | -- | GLN 249 |
| LEU 250 | LEU 250 | LEU 250 |
| ILE 251 | ILE 251 | -- |
| ASN 252 | ASN 252 | ASN 252 |
| 1-PDB ID 6J17 refer to experimental data^1^. * kcal/mol refer to ΔG molecular docking evaluations. | | |
