# Supplementary material for: Mycobacterium susceptibility to ivermectin by inhibition of eccD3, an ESX-3 secretion system component
Source: PLoS Comput Biol. 2025 Apr 17;21(4):e1012936. doi: 10.1371/journal.pcbi.1012936 (PMC12005495; doi:10.1371/journal.pcbi.1012936)
Supplement: S4 Table — (DOCX) [file pcbi.1012936.s016.docx]

S4 Table. Molecular docking lowest binding energy and Z-scores for antituberculosis drugs on ESX-3 secretion system components

| **Drugs** | **Binding energy ΔG kcal/mol / Z-score** | | | | | | | | | | | |
| --- | --- | --- | --- | --- | --- | --- | --- | --- | --- | --- | --- | --- |
|  | **EccB3** | | **EccD3 N-ter** | | **EccD3 C-ter** | | **ESX-3 P1** | | **ESX-3 P2** | | **EccC3 ATPase domain III** | |
|  | kca/  mol | Z-score | kcal/ mol | Z-score | kcal/  mol | Z-score | kcal/  mol | Z-score | kcal/  mol | Z-score | kcal/  mol | Z-score |
| Isoniazid | -4.90 | 1.19 | -4.00 | 1.06 | -4.80 | 1.08 | -6.1 | 0.76 | -5.7 | 1.06 | -5.0 | 1.22 |
| Pyrazinamide | -4.60 | 1.41 | -3.70 | 1.30 | -4.20 | 1.46 | -5.2 | 1.43 | -4.9 | 1.63 | -5.1 | 1.12 |
| Ethambutol | -4.10 | 1.78 | -3.40 | 1.54 | -4.10 | 1.52 | -5.0 | 1.58 | -5.0 | 1.56 | -4.1 | 2.06 |
| Rifampicin | -7.10 | -0.42 | -6.70 | -1.11 | -6.90 | -0.26 | -7.8 | -0.49 | -7.7 | -0.34 | -6.4 | -0.09 |
| Rifanpentine | -7.90 | -1.00 | -7.50 | -1.75 | -7.80 | -0.83 | -8.3 | -0.86 | -8.2 | -0.70 | -7.0 | -0.65 |
| Rifalazil | -8.10 | -1.15 | -6.20 | -0.71 | -8.10 | -1.02 | -8.4 | -0.94 | -8.6 | -0.98 | -6.6 | -0.28 |
| Rifabutin | -7.60 | -0.78 | -6.00 | -0.55 | -6.80 | -0.19 | -8.3 | -0.86 | -7.9 | -0.48 | -6.0 | 0.28 |
| Amikacin | -6.20 | 0.24 | -4.30 | 0.82 | -5.80 | 0.44 | -6.9 | 0.17 | -6.9 | 0.22 | -6.9 | -0.56 |
| Streptomycin | -6.70 | -0.12 | -4.60 | 0.57 | -6.90 | -0.26 | -7.9 | -0.57 | -7.8 | -0.41 | -6.8 | -0.46 |
| kanamycin | -6.00 | 0.39 | -5.10 | 0.17 | -6.00 | 0.31 | -7.8 | -0.49 | -7.1 | 0.08 | -6.6 | -0.28 |
| Levofloxacin | -7.80 | -0.93 | -5.70 | -0.31 | -7.00 | -0.32 | -7.9 | -0.57 | -8.2 | -0.70 | -7.4 | -1.03 |
| Sparfloxacin | -7.90 | -1.00 | -5.40 | -0.07 | -7.70 | -0.77 | -7.5 | -0.27 | -8.0 | -0.56 | -6.2 | 0.10 |
| Pazufloxacin | -7.50 | -0.71 | -5.50 | -0.15 | -6.70 | -0.13 | -7.6 | -0.35 | -8.0 | -0.56 | -6.8 | -0.46 |
| Ethionamide | -4.70 | 1.34 | -3.80 | 1.22 | -4.70 | 1.14 | -5.6 | 1.13 | -5.2 | 1.42 | -4.8 | 1.41 |
| Pretomanid | -6.80 | -0.20 | -6.00 | -0.55 | -7.10 | -0.39 | -7.2 | -0.05 | -7.8 | -0.41 | -7.3 | -0.93 |
| Bedaquiline | -7.30 | -0.56 | -6.00 | -0.55 | -7.90 | -0.89 | -7.7 | -0.42 | -7.0 | 0.15 | -6.4 | -0.09 |
| Linezolid | -6.90 | -0.27 | -5.60 | -0.23 | -7.10 | -0.39 | -7.2 | -0.05 | -7.5 | -0.20 | -6.6 | -0.28 |
| Aminosalicylic acid | -5.20 | 0.97 | -4.40 | 0.74 | -4.90 | 1.01 | -6.1 | 0.76 | -5.7 | 1.06 | -5.6 | 0.66 |
| Cycloserine | -4.00 | 1.85 | -3.20 | 1.70 | -3.30 | 2.03 | -4.4 | 2.02 | -4.5 | 1.91 | -4.2 | 1.97 |
| Amithiozone | -5.80 | 0.53 | -4.50 | 0.65 | -5.90 | 0.38 | -5.9 | 0.91 | -5.9 | 0.92 | -5.2 | 1.03 |
| Thiosulfuric acid | -3.40 | 2.29 | -2.70 | 2.10 | -2.70 | 2.41 | -3.4 | 2.76 | -3.6 | 2.54 | -3.9 | 2.25 |
| Viomycin | -7.60 | -0.78 | -5.50 | -0.15 | -7.50 | -0.64 | -8.8 | -1.23 | -7.9 | -0.48 | -6.6 | -0.28 |
| Enviomycin | -7.20 | -0.49 | -5.20 | 0.09 | -6.00 | 0.31 | -7.6 | -0.35 | -8.8 | -1.12 | -7.8 | -1.40 |
| Florfenicol | -6.00 | 0.39 | -4.80 | 0.41 | -6.10 | 0.25 | -6.0 | 0.84 | -7.2 | 0.01 | -6.6 | -0.28 |
| Vanoxerine | -6.90 | -0.27 | -6.20 | -0.71 | -7.80 | -0.83 | -8.0 | -0.64 | -8.7 | -1.05 | -6.7 | -0.37 |
| Metformin | -4.80 | 1.27 | -3.70 | 1.30 | -4.10 | 1.52 | -5.8 | 0.99 | -5.3 | 1.35 | -5.6 | 0.66 |
| Vitamin D | -6.60 | -0.05 | -6.40 | -0.87 | -8.10 | -1.02 | -7.4 | -0.20 | -8.7 | -1.05 | -6.3 | 0.00 |
| Simvastatin | -6.70 | -0.12 | -5.40 | -0.07 | -7.70 | -0.77 | -7.3 | -0.12 | -8.1 | -0.63 | -6.9 | -0.56 |
| Tamoxifen | -6.50 | 0.02 | -5.50 | -0.15 | -6.60 | -0.07 | -7.0 | 0.10 | -7.5 | -0.20 | -6.1 | 0.19 |
| Fluvastatin | -7.00 | -0.34 | -5.70 | -0.31 | -7.10 | -0.39 | -7.4 | -0.20 | -8.0 | -0.56 | -7.1 | -0.75 |
| Avermectin | -7.30 | -0.56 | -6.40 | -0.87 | -8.50 | -1.28 | -8.8 | -1.23 | -8.3 | -0.77 | -7.0 | -0.65 |
| Ivermectin | -9.00 | -1.81 | -6.80 | -1.19 | -8.10 | -1.02 | -8.7 | -1.16 | -8.6 | -0.98 | -7.0 | -0.65 |
| Moxidectin | -7.80 | -0.93 | -7.50 | -1.75 | -8.40 | -1.21 | -8.7 | -1.16 | -8.4 | -0.84 | -8.8 | -2.33 |
| Selamectin | -8.10 | -1.15 | -7.40 | -1.67 | -8.40 | -1.21 | -8.8 | -1.23 | -8.5 | -0.91 | -6.9 | -0.56 |
| ATP | ND | ND | ND | ND | ND | ND | ND | ND | ND | ND | -9.0 | ND |
| ND, not determined; EccD3 N-ter, EccD3 N-terminal; EccD3 C-ter, EccD3 C-terminal; ESX-3 P1 and ESX-3 P2 correspond to ESX-3 protomer 1 and 2, respectively. | | | | | | | | | | | | |
