# Supplementary material for: Mycobacterium susceptibility to ivermectin by inhibition of eccD3, an ESX-3 secretion system component
Source: PLoS Comput Biol. 2025 Apr 17;21(4):e1012936. doi: 10.1371/journal.pcbi.1012936 (PMC12005495; doi:10.1371/journal.pcbi.1012936)
Supplement: S5 Table — (DOCX) [file pcbi.1012936.s017.docx]

S5 Table. Avermectin drugs properties.

|  | **Molecular weight** | **Hydrogen bond acceptors** | **Hydrogen bond donors** | **LogP** | **Rotable Bond count** | **Solubility** |
| --- | --- | --- | --- | --- | --- | --- |
| Avermectin | 873.1 g/mol | 14 | 3 | 2.31 | 8 | -3.42 |
| Ivermectin | 875.1 g/mol | 14 | 3 | 2.72 | 8 | -3.89 |
| Moxidectin | 639.8 g/mol | 9 | 2 | 4.16 | 3 | -4.37 |
| Selamectin | 770.0 g/mol | 12 | 3 | 3.09 | 4 | -4.07 |
