# Supplementary material for: Mycobacterium susceptibility to ivermectin by inhibition of eccD3, an ESX-3 secretion system component
Source: PLoS Comput Biol. 2025 Apr 17;21(4):e1012936. doi: 10.1371/journal.pcbi.1012936 (PMC12005495; doi:10.1371/journal.pcbi.1012936)
Supplement: S6 Table — (DOCX) [file pcbi.1012936.s018.docx]

S6 Table. Molecular docking lowest binding energy for the 34 drugs and EccD3-hydrophobix and EccE3-hydrophilic non interface regions (*M. tuberculosis*).

| **Binding energy** | | | | | | |
| --- | --- | --- | --- | --- | --- | --- |
| **Drugs** | **EccD3** | | | **EccE3** | | |
|  | **Min** | **Mean** | **SD** | **Min** | **Mean** | **SD** |
|  | ΔG Kcal/mol | |  | ΔG kcal/mol | |  |
| Isoniazid | -4.6 | -4.6 | 9.3622E-16 | -5.3 | -4.68 | 0.31552426 |
| Pyrazinamide | -5 | -4.1 | 0.31622777 | -4.4 | -3.97 | 0.24966644 |
| Ethambutol | -4.2 | -4.2 | 9.3622E-16 | -5.5 | -4.94 | 0.59104427 |
| Rifampicin | -7.5 | -7.5 | 0 | -6.6 | -6.6 | 0 |
| Rifanpentine | -8 | -8 | 0 | -7.4 | -7.06666667 | 0.31622777 |
| Rifalazil | -8.2 | -8.2 | 1.8724E-15 | -7.4 | -7.24 | 0.21186998 |
| Rifabutin | -8 | -8 | 0 | -6.4 | -6.32 | 0.06324555 |
| Amikacin | -5.5 | -5.44 | 0.05163978 | -6.2 | -6.03 | 0.06749486 |
| Streptomycin | -5.5 | -5.45 | 0.07071068 | -6.3 | -6.09 | 0.23309512 |
| kanamycin | -5.4 | -5.4 | 9.3622E-16 | -6.6 | -6.55 | 0.05270463 |
| Levofloxacin | -6.9 | -6.81 | 0.03162278 | -6.5 | -6.17 | 0.15670212 |
| Sparfloxacin | -6.5 | -6.5 | 0 | -6.2 | -6.2 | 9.3622E-16 |
| Pazufloxacin | -6.2 | -6.2 | 9.3622E-16 | -5.8 | -5.77 | 0.04830459 |
| Ethionamide | -4.3 | -4.3 | 9.3622E-16 | -4.4 | -4.15 | 0.14337209 |
| Pretomanid | -6.6 | -6.56 | 0.05163978 | -6.4 | -6.28 | 0.09189366 |
| Bedaquiline | -6.7 | -6 | 0.37712362 | -6.9 | -6.4 | 0.2981424 |
| Linezolid | -6.5 | -6.4 | 0.06666667 | -6 | -5.89 | 0.12866839 |
| Aminosalicylic acid | -4.8 | -4.8 | 9.3622E-16 | -4.9 | -4.74 | 0.21186998 |
| Cycloserine | -4.6 | -4.18 | 0.30110906 | -4.3 | -4.27 | 0.04830459 |
| Amithiozone | -5 | -4.93 | 0.06749486 | -5.6 | -5.04 | 0.36878178 |
| Thiosulfuric acid | -3.7 | -3.69 | 0.03162278 | -3.4 | -3.4 | 4.6811E-16 |
| Viomycin | -6.4 | -6.39 | 0.03162278 | -6.6 | -6.6 | 0 |
| Enviomycin | -6.8 | -6.77 | 0.04830459 | -6.3 | -6.09 | 0.22335821 |
| Florfenicol | -5.4 | -5.31 | 0.03162278 | -5.3 | -5.2 | 0.06666667 |
| Vanoxerine | -7.4 | -6.99 | 0.23309512 | -6.9 | -6.58 | 0.22509257 |
| Metformin | -4.1 | -4.1 | 9.3622E-16 | -4.7 | -4.4 | 0.31622777 |
| Vitamin D | -6.9 | -6.35 | 0.33082389 | -6.2 | -6.14 | 0.09660918 |
| Simvastatin | -6.9 | -6.45 | 0.1779513 | -6.5 | -6.5 | 0 |
| Tamoxifen | -6.5 | -6.44 | 0.06992059 | -5.8 | -5.69 | 0.05676462 |
| Fluvastatin | -6.7 | -6.53 | 0.21628171 | -6.5 | -6.3 | 0.2 |
| Avermectin | -3.4 | -3.44 | 0.23309512 | -5.2 | -5.14 | 0.09660918 |
| Ivermectin | -3.3 | -3.3 | 9.3622E-16 | -5.5 | -5.33 | 0.18287822 |
| Moxidectin | -3.5 | -3.45 | 0.07071068 | -5.3 | -5.2 | 0.06666667 |
| Selamectin | -3.4 | -3.4 | 9.3622E-16 | -5.8 | -5.69 | 0.05676462 |
| Min- lowest binding energy, Mean, and (SD) standard deviation of the 10 times docking protein-drug simulations. | | | | | | |
