# Supplementary material for: Mycobacterium susceptibility to ivermectin by inhibition of eccD3, an ESX-3 secretion system component
Source: PLoS Comput Biol. 2025 Apr 17;21(4):e1012936. doi: 10.1371/journal.pcbi.1012936 (PMC12005495; doi:10.1371/journal.pcbi.1012936)
Supplement: S7 Table — (DOCX) [file pcbi.1012936.s019.docx]

S7 Table. Student´s t-test *results* between *M. smegmatis* PLJR962-*eccD3*-gRNA and *M. smegmatis* PLJR962-*eccD3*-gRNA ATc in presence of 64 µg/mL and 32 µg/mL ivermectin.

| **Ivermectin** | | | | |
| --- | --- | --- | --- | --- |
| **64 µg/mL** | | | **32 µg/mL** | |
| **Hr.** | ***p* value** | ***t*-stadistic** | ***p* value** | ***t*-stadistic** |
| 20 | 0.000531758 | 10.1438473 | 0.03136107 | 3.25049447 |
| 21 | 0.000373251 | 11.111275 | 0.00037325 | 11.111275 |
| 22 | 0.000670005 | 9.5554851 | 0.00067001 | 9.5554851 |
| 23 | 0.000369393 | 11.1409474 | 0.00036939 | 11.1409474 |
| 24 | 0.000614336 | 9.77248833 | 0.00061434 | 9.77248833 |
| 25 | 0.000230415 | 12.5714286 | 0.00023042 | 12.5714286 |
| 26 | 0.000401188 | 10.9071806 | 0.00040119 | 10.9071806 |
| 27 | 8.32527E-05 | 16.2827488 | 0.00071827 | 9.38470086 |
| Critical *t*-value (two tail) 2.77644511 | | | | |
