# Supplementary material for: Mycobacterium susceptibility to ivermectin by inhibition of eccD3, an ESX-3 secretion system component
Source: PLoS Comput Biol. 2025 Apr 17;21(4):e1012936. doi: 10.1371/journal.pcbi.1012936 (PMC12005495; doi:10.1371/journal.pcbi.1012936)
Supplement: S8 Table — (DOCX) [file pcbi.1012936.s020.docx]

S8 Table. Student´s t-test *results* between *M. smegmatis* PLJR962-*eccD3*-gRNA and *M. smegmatis* PLJR962-*eccD3*-gRNA ATc in presence of 0.5 µg/mL linezolid and 0.8 µg/mL rifampicin.

| **Linezolid**  **0.5 µg/mL** | | | **Rifampicin**  **0.8 µg/mL** | |  |
| --- | --- | --- | --- | --- | --- |
| **Hr.** | ***p* value** | ***t*-stadistic** | ***p* value** | ***t*-stadistic** |  |
| 20 | 0.01764102 | 3.89331411 | 0.0024247 | -6.81385144 |  |
| 21 | 0.01931456 | 3.78725278 | 0.00133821 | -7.97741172 |  |
| 22 | 0.0063787 | 5.23114374 | 0.00270543 | -6.61649917 |  |
| 23 | 0.00333066 | 6.25543242 | 0.00192125 | -7.2502025 |  |
| 24 | 0.00312188 | 6.36600142 | 0.00201784 | -7.15625 |  |
| 25 | 0.00291983 | 6.48203724 | 0.00210018 | -7.08044159 |  |
| 26 | 0.0037544 | 6.05515071 | 0.00151133 | -7.72581418 |  |
| 27 | 0.00436816 | 5.80963207 | 0.00153675 | -7.69188463 |  |
| Critical *t*-value (two tail) 2.77644511 | | | | | |
