# Supplementary material for: Mycobacterium susceptibility to ivermectin by inhibition of eccD3, an ESX-3 secretion system component
Source: PLoS Comput Biol. 2025 Apr 17;21(4):e1012936. doi: 10.1371/journal.pcbi.1012936 (PMC12005495; doi:10.1371/journal.pcbi.1012936)
Supplement: S9 Table — (DOCX) [file pcbi.1012936.s021.docx]

S9 Table. Avermectin drugs biological activities.

| **Biological activity drugs** | **Pa value** | | | |
| --- | --- | --- | --- | --- |
|  | **Avermectin** | **Ivermectin** | **Moxidectin** | **Selamectin** |
| Insecticide | 0.990 | 0.989 | 0.959 | 0.956 |
| Antiparasitic | 0.994 | 0.994 | 0.982 | 0.949 |
| Acaricide | 0.978 | 0.971 | 0.941 | 0.939 |
| Antihelmintic | 0.994 | 0.994 | 0.984 | 0.931 |
| Antifungal | 0.915 | 0.929 | 0.875 | 0.880 |
| Antibacterial | 0.919 | 0.921 | 0.813 | 0.857 |
| Antibiotic | 0.894 | 0.893 | 0.734 | 0.787 |
| Pa value represents probability to be active. Biological activities were determined by PASSonline | | | | |
