# Supplementary material for: Mycobacterium susceptibility to ivermectin by inhibition of eccD3, an ESX-3 secretion system component
Source: PLoS Comput Biol. 2025 Apr 17;21(4):e1012936. doi: 10.1371/journal.pcbi.1012936 (PMC12005495; doi:10.1371/journal.pcbi.1012936)
Supplement: S10 Table — (DOCX) [file pcbi.1012936.s022.docx]

S10 Table. Avermectin drugs toxicity.

| **Biological activity drugs** | **Toxicity** | | | |
| --- | --- | --- | --- | --- |
|  | **Avermectin** | **Ivermectin** | **Moxidectin** | **Selamectin** |
| LD50 | 19 mg/kg | 27 mg/kg | 310 mg/kg | 310 mg/kg |
| Hepatotoxicity | 0.85-I | 0.93-I | 0.69-I | 0.67-I |
| Carcinogenicity | 0.64-I | 0.66-I | 0.54-I | 0.52-I |
| Mutagenicity | 0.65-I | 0.89-I | 0.63-I | 0.67-I |
| Cytotoxicity | 0.70-I | 0.72-I | 0.68-I | 0.76-I |
| Immunotoxicity | 0.99-A | 0.99-A | 0.99-A | 0.99-A |
| A and I suffixes represent active and inactive activity probability, respectively. LD50, lethal dose at 50%. Toxicity analyses were obtained from Pro Tox-II. | | | | |
