# Supplementary material for: Mycobacterium susceptibility to ivermectin by inhibition of eccD3, an ESX-3 secretion system component
Source: PLoS Comput Biol. 2025 Apr 17;21(4):e1012936. doi: 10.1371/journal.pcbi.1012936 (PMC12005495; doi:10.1371/journal.pcbi.1012936)
Supplement: S11 Table — (DOCX) [file pcbi.1012936.s023.docx]

S11 Table. Avermectin drugs ADME properties.

| **Biological activity drugs** | **ADME properties** | | | | |
| --- | --- | --- | --- | --- | --- |
|  | **Avermectin** | **Ivermectin** | **Moxidectin** | **Selamectin** | |
| Water solubility Log S  (SILICOs-IT) | -3.42  soluble | -3.89  soluble | -4.37 moderately soluble | -4.07 moderately soluble | |
| Lipophilicity Log P O/W | 2.31 | 2.72 | 4.16 | 3.09 | |
| GI absorption | Low | Low | Low | Low | |
| BBB permeability | No | No | No | No | |
| P-glycoprotein substrate | Yes | Yes | Yes | Yes | |
| Skin permeation Log Kp | -8.90 cm/s | -7.14 cm/s | -5.59 cm/s | -7.13 cm/s | |
| BBB, blood brain barrier; GI, gastrointestinal. Log S, solubility logarithm. Log P O/W, logarithm of the n-octanol/water partition coefficient. Log Kp, skin permeation coefficient logarithm. SILICOs-IT, hybrid fragmental/topological method calculated by FILTER-IT program. ADME properties were obtained from SwissADME. | | | | |  |
