# Supplementary material for: Mycobacterium susceptibility to ivermectin by inhibition of eccD3, an ESX-3 secretion system component
Source: PLoS Comput Biol. 2025 Apr 17;21(4):e1012936. doi: 10.1371/journal.pcbi.1012936 (PMC12005495; doi:10.1371/journal.pcbi.1012936)
Supplement: S12 Table — (DOCX) [file pcbi.1012936.s024.docx]

S12 Table. Identity percent of the alignment between ESX3 secretion system nucleotide sequence *M. smegmatis* and *M. tuberculosis*.

| **ESX3 cluster** | **NCBI ID** | **Coordinates** | **Length** | **Identity percent** |
| --- | --- | --- | --- | --- |
| *M. smegmatis* | CP000480.1 | 691,998 – 706,477 | 14, 480 bp | 66.8% |
| *M. tuberculosis* | NC_000962.3 | 342,130 – 356,875 | 14, 746 bp |  |
