# Supplementary material for: Mycobacterium susceptibility to ivermectin by inhibition of eccD3, an ESX-3 secretion system component
Source: PLoS Comput Biol. 2025 Apr 17;21(4):e1012936. doi: 10.1371/journal.pcbi.1012936 (PMC12005495; doi:10.1371/journal.pcbi.1012936)
Supplement: S13 Table — (DOCX) [file pcbi.1012936.s025.docx]

S13 Table. Identity percent of the alignment between *eccD3* gene nucleotide sequence *M. smegmatis* and *M. tuberculosis*.

| ***eccD3* gene** | **NCBI ID** | **Coordinates** | **Length** | **Identity percent** |
| --- | --- | --- | --- | --- |
| *M. smegmatis* | CP000480.1 | 702,737 – 704,164 | 1,427 bp | 68.4% |
| *M. tuberculosis* | NC_000962.3 | 353,083 – 354,501 | 1,418 bp |  |
