# Supplementary material for: Mycobacterium susceptibility to ivermectin by inhibition of eccD3, an ESX-3 secretion system component
Source: PLoS Comput Biol. 2025 Apr 17;21(4):e1012936. doi: 10.1371/journal.pcbi.1012936 (PMC12005495; doi:10.1371/journal.pcbi.1012936)
Supplement: S14 Table — (DOCX) [file pcbi.1012936.s026.docx]

S14 Table. Sampling box center and dimensions for each protein used for the molecular docking.

| **Protein model** | **Grid center**  **(x, y, z)** | **Size**  **Å** |
| --- | --- | --- |
| EccB3 | 163.052, 160.002, 144.649 | 36.4192, 49.7789, 48.8539 |
| EccD3 N-terminal | 215.316, 208.195, 196.713 | 25.676, 23.5991, 41.3019 |
| EccD3 C-terminal | 217.127, 178.081, 181.243 | 26.1652, 32.091, 42.0759 |
| ATPase domain III of EccC3 | 22.59, -7.20, 23.23 | 30.00, 33.00, 30.00 |
| ESX-3 protomer 1 | 159.167, 162.145, 197.592 | 44.9949, 49.4289, 48.4317 |
| ESX-3 protomer 2 | 189.304, 172.168, 210.911 | 56.6132, 42.4863, 41.0964 |
