# Supplementary material for: Mycobacterium susceptibility to ivermectin by inhibition of eccD3, an ESX-3 secretion system component
Source: PLoS Comput Biol. 2025 Apr 17;21(4):e1012936. doi: 10.1371/journal.pcbi.1012936 (PMC12005495; doi:10.1371/journal.pcbi.1012936)
Supplement: S15 Table — (DOCX) [file pcbi.1012936.s027.docx]

S15 Table. Primers and probes used in this study.

| **Amplifying region** | **Primer Forward 5’-3’** | **Primer Reverse 5’-3’** | **Probe 5’-3’ NFQ** |
| --- | --- | --- | --- |
| *eccD3*-gRNA  182 bp | Primer *eccD3*-gRNA  ACCCCACTGGCGCCTGC | Primer Sec 1834  TTCCTGTGAAGAGCCATTGA | N/A |
| *eccD3* region | GTCGCCGCGTGGTC | CGGTGAACACCGCGATAC | GTCGCCGCGTGGTCGTTGATCAGCA |
| *sigA* region | CGCCTCCAGCAGATGGTTTT | CGAGAAGGGCGAGAAGCT | GCATGTCGCGGCGCTGCTGCACTGG |
